# Revealing mechanisms of mating plug function under sexual selection

**DOI:** 10.1073/pnas.1920526117

**Published:** 2020-10-19

**Authors:** Paula Stockley, Catarina Franco, Amy J. Claydon, Amanda Davidson, Dean E. Hammond, Philip J. Brownridge, Jane L. Hurst, Robert J. Beynon

**Affiliations:** ^a^Mammalian Behaviour and Evolution Group, Institute of Infection, Veterinary and Ecological Sciences, University of Liverpool, CH64 7TE Neston, United Kingdom;; ^b^Centre for Proteome Research, Institute of Systems, Molecular and Integrative Biology, University of Liverpool, L69 7ZB Liverpool, United Kingdom

**Keywords:** reproduction, ejaculates, sexual selection, proteomics, stable isotope labeling

## Abstract

Mating plugs are produced by many sexually reproducing animals and are hypothesized to promote male fertilization success under promiscuous mating. However, tests of this hypothesis have been constrained by an inability to discriminate ejaculates of different males in direct competition. Here, we use stable isotope labeling in vivo and proteomics to achieve this in a promiscuous rodent, *Myodes glareolus*. We show that, although the first male’s plug is usually dislodged, it can be retained throughout the second male’s copulation. Retained plugs did not completely block rival sperm but did significantly limit their numbers. Differences in the number of each male’s sperm progressing through the female reproductive tract were also explained by natural variation in the size of mating plugs and reproductive accessory glands from which major plug proteins originate. Relative sperm numbers in turn predicted the relative fertilization success of rival males. Our application of stable isotopes to label ejaculates resolves a longstanding debate by revealing how rodent mating plugs promote fertilization success under competitive conditions. This approach opens new opportunities to reveal cryptic mechanisms of postcopulatory sexual selection among diverse animal taxa.

## Significance

Promiscuous mating by females leads to competition between males for fertilization success. When fertilization is internal, this means that rival males’ sperm must compete within the female reproductive tract to reach the eggs. Males of diverse species deposit a mating plug during copulation, which is hypothesized to assist in the race for fertilization following multiple mating. Here, we tested this by using stable isotope labeling to discriminate the ejaculates of competing male voles in direct competition. This revealed that the mating plug simultaneously inhibits the sperm of rival males while promoting transport of a male’s own sperm, both of which are beneficial in the competition for fertilizations.

Mating plugs are produced by diverse sexually reproducing animals, from worms to primates (1–5). Typically, plugs are formed by coagulation of ejaculated proteins to form a solid mass within the female reproductive tract (2, 5), and speculation as to their function spans three centuries of scientific research (2, 4, 5). Early investigators hypothesized a necessary role of the plug for successful reproduction (2, 5), shaped by natural selection. However, following the discovery that females often mate sequentially with more than one male, emphasis has shifted to the (nonmutually exclusive) idea that mating plug function has evolved under sexual selection (2, 5, 6). That is, when females mate promiscuously, plugs may serve to block the sperm of rival males or to facilitate transport of a male’s own sperm. Either or both potential mechanisms could enhance a male’s reproductive success under sperm competition (2), potentially favoring the evolution of larger or firmer plugs that are more difficult for rival males or recently mated females to dislodge (5–7).

**Fig. 1.**
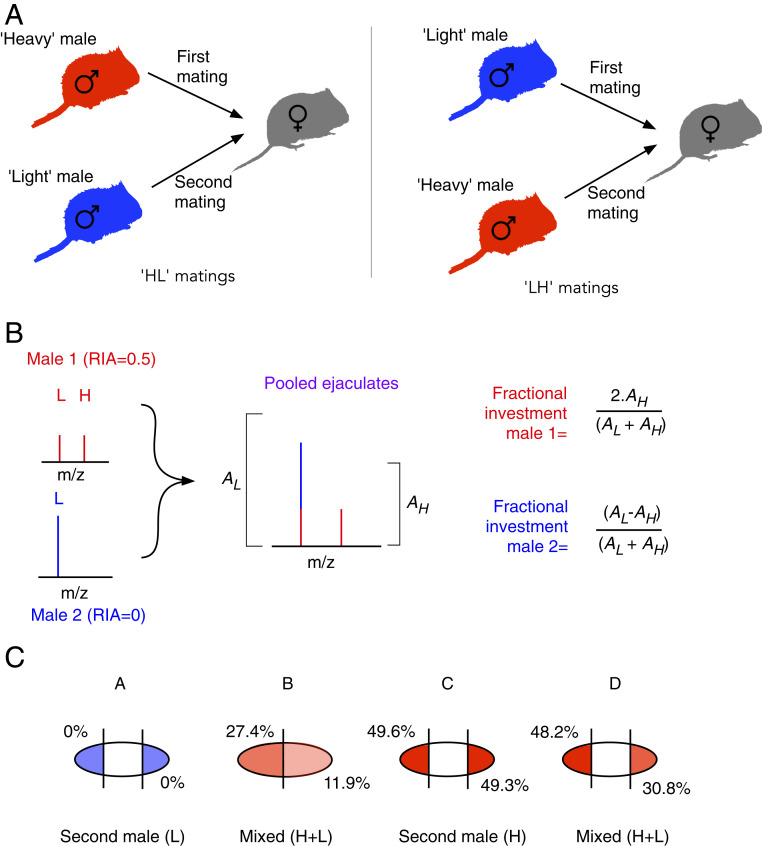
Evidence for dislodgment and retention of mating plugs following sequential copulations. (*A*) Mating sequences. Plugs were recovered from female bank voles immediately after sequential copulation with two males, differentially labeled with stable isotopes, during which each male ejaculated once. (*B*) Determining the origin of mating plugs. Mating plugs were digested with trypsin and analyzed by LC-MS/MS. To assess the origin of the plugs (first male, second male, or both), the isotope profile of the peptide SASGSSTSYSLDK was analyzed according to the panel in the figure. (*C*) Exemplar mating plugs. In most cases (13 of 17, examples in A and C), proteomic analysis revealed that the recovered plug material originated entirely from the second male to mate: When the second male was “light,” the plug contained no [^13^C_6_] lysine (example in A), and, when the second male was “heavy,” the plug contained 50% [^13^C_6_] lysine (example in C), consistent with 0.5 labeling. However, in some cases (4 of 17, examples in B and D), proteomic analysis revealed that recovered plug material originated from both the first and second males to mate. Further details of all plug samples analyzed are provided in *SI Appendix*, Figs. S2 and S7.

The solid mating plug produced by rodents has been a subject of particular interest (5, 6, 8, 9), with several sexually selected functions hypothesized. Rodent mating plugs form in the female reproductive tract when seminal vesicle-derived proteins in the ejaculate cross-link and coagulate in the presence of a prostate-derived transglutaminase, TGM4 (5, 8). A role for the plug in sperm competition, either as a mechanical barrier to rival sperm or in promoting transport of a male’s own sperm, is often suggested (5, 6, 8). However, evidence to distinguish plug functions is indirect (8, 10) or derived from experiments involving extreme manipulations, such as removal of male accessory glands or gene knock-out (e.g., refs. 11–13). Although such tests provide useful findings, the complete removal or significant disruption of processes leading to the formation of mating plugs may be detrimental to normal reproductive function, therefore providing limited insights as to the selection pressures acting on plug function under competitive conditions. To determine the role of mating plugs in competition therefore requires quantifying how their naturally occurring variation affects male fitness. However, direct tests of the mechanistic function of mating plugs under naturalistic conditions have been constrained by an inability to discriminate the ejaculates of different males within the female reproductive tract following multiple mating. Here, we investigate the role of rodent mating plugs under a typical sperm competition scenario where a female mates sequentially with two males. By employing differential stable isotope labeling and proteomics (14, 15), we are able to distinguish the proteins present in competing ejaculates (Fig. 1 *A* and *B*) and quantify the consequences of natural variation in mating plug retention and size on sperm numbers progressing through the female reproductive tract. This proteomic labeling approach reveals insight into mating plug function in a model promiscuous rodent, the bank vole (*Myodes glareolus*).

## Results and Discussion

**Fig. 2.**
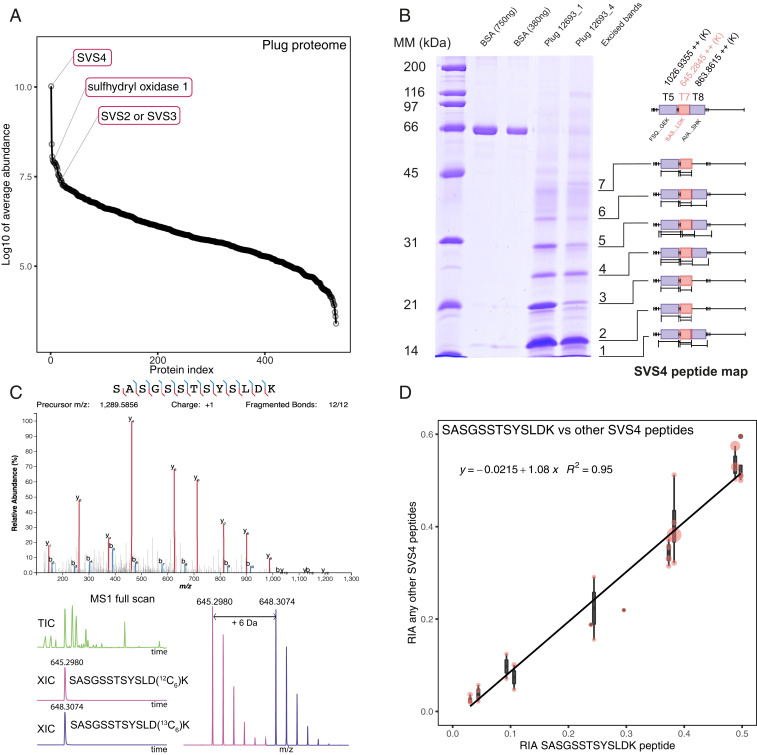
Assessment of mating plugs by peptide mass spectrometry. When plugs were analyzed by proteomics, over 500 proteins were identified. However, the abundance distribution (assessed by label-free quantification) indicates that one protein in particular, SVS4, was over two orders of magnitude more abundant than other proteins (*A*). Mating plugs from bank voles exhibit a simple pattern on SDS/PAGE, consistent with a polymeric series of a low molecular weight protein (*B*). This was confirmed by in-gel digests—relevant shaded, boxed peptides from each gel band confirm the primary protein is SVS4 (*B*). One peptide, SASGSSTSYSLDK (pink in *B*), confirmed by a complete set of product ions, yielded very strong signals and low noise mass spectra in every plug sample, permitting accurate assignment of plug origin (*C*). Although this “index” peptide was the strongest ion and was seen in every plug sample (probably because of the absence of cross-linking sites), other peptides in SVS4 were also measured, and the calculation of RIA from those peptides, relative to the index peptide (*D*), was highly correlated (see also *SI Appendix*, Fig. S1). Abbreviations: MM, molecular weight markers; BSA, bovine serum albumin; TIC, total ion chromotogram; XIC, extracted ion chromatogram.

A key challenge was the quantification of the contribution of two different males, in terms of the mating plug or sperm numbers. Following sequential copulations with two males, our goals were to determine: 1) the origin of the mating plug(s) remaining in the female reproductive tract (first male, second male, or both), and 2) to quantify sperm numbers of each male reaching the uterus. To achieve this, one male of each competing pair was labeled with a diet in which half of the lysine was presented in stable isotope form, labeled with six carbon-13 atom centers ([^13^C_6_]lysine). Stable isotope-labeled amino acids have the same chemical properties as the natural carbon-12 form and thus behave in the same way and are distinguishable only by mass spectrometry. By feeding voles this diet, the relative contributions of two males were readily assessed. The proteins in the ejaculate were analyzed by proteomics methodologies, and peptides terminated with a lysine residue and containing one instance of the labeled amino acid were reliable indicators of a relative contribution. To identify which male(s) had produced each plug, it was necessary to find peptides from the highly abundant proteins in the solidified, polymerized coagulation plug. Analysis of the coagulation plug proteome, either by global proteomic analysis or by analysis of bands on protein-resolved sodium dodecyl sulfate polyacrylamide gel electrophoresis (SDS/PAGE), revealed that by far the most abundant protein was SVS4 (Fig. 2 *A* and *B*), over 100 times more abundant than the next most likely seminal vesicle protein. Further, because this is cross-linked heavily to stabilize and make the plug insoluble, there were relatively few peptides that could be used. One peptide in particular (SASGSSTSYSLDK, pink boxes in Fig. 2*B*) was present in every sample, in high abundance, permitting accurate recovery of the distribution between labeled and unlabeled forms (Fig. 2*C*). Moreover, this peptide was representative of the isotope distribution of all other peptides that were only present in a limited subset of samples (Fig. 2*D* and *SI Appendix*, Fig. S1) and was thus suitable on multiple counts for determining the origin of the plug.

Previous observations in the bank vole suggest that the mating plug deposited by a female’s first mate is likely to be dislodged prior to ejaculation by a second male, a process that is facilitated by repeated intromissions during copulation (16). Consistent with this expectation, we found that, in most cases (13 of 17, ∼75%), the mating plug recovered after sequential copulation with two males originated entirely from the second male (Fig. 1 *C*, *A* and *C*). However, in some cases (4 of 17, ∼25%), labeling revealed that plug material from the first male remained within the female reproductive tract after a second male had ejaculated (Fig. 1 *C*, *B* and *D*). This resulted in “double plugs” (plugs originating from sequential ejaculations that have become bound together), containing a gradient of proteins from two different males (Fig. 1 *C*, *D*). Where double plugs split into two on recovery, the section closest to the cervix contained plug protein from the first male combined with protein from the second male (Fig. 1 *C*, *B* and *SI Appendix*, Fig. S2) whereas the plug section behind this contained protein only from the second male (*SI Appendix*, Fig. S2). Hence, the first male’s plug was retained closer to the cervix.

**Fig. 3.**
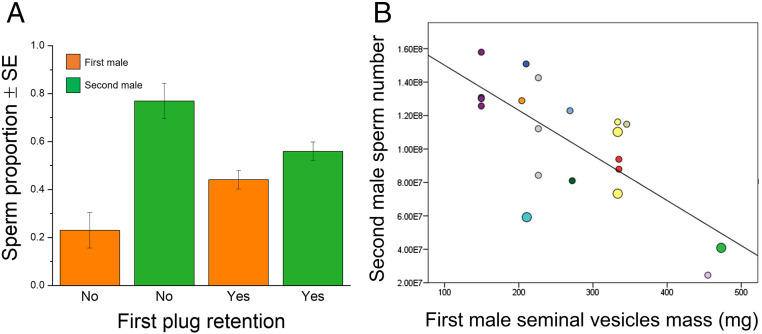
Influence of mating plugs on the number of sperm from competing males reaching the uterus. (*A*) When two males mated with the same female, the relative proportion of their sperm reaching the uterus differed according to whether or not the first male’s mating plug was retained. For statistical analysis, see Table 1. (*B*) When two males mated with the same female, fewer of the second male’s sperm reached the uterus when the first male to mate had larger seminal vesicles. Different colored points are used to distinguish 12 males that mated first in 19 double copulations, each with different competitors. Larger points indicate where the plug recovered after the double copulation consisted of proteins derived from both males. For statistical analysis, see A in Table 3.

Unexpectedly, given that rodent plugs typically adhere firmly to the vaginal epithelium (6, 17), we found that retained first male plugs did not completely block rival sperm. Rather, sperm from a second male were still able to reach the uterus when plug material from a first male was present in the female reproductive tract (Fig. 3*A* and *SI Appendix*, Fig. S3*A*). However, our data reveal that retention of the first male’s plug significantly influenced the relative number of competing males’ sperm reaching the uterus (Fig. 3*A* and Table 1). That is, when the first male’s plug was retained, relatively fewer sperm from the second male’s ejaculate were recovered from the uterus. This result appears to be largely driven by inhibited progression of the second male’s sperm, with fewer absolute numbers reaching the uterus when the first male’s plug is retained (*SI Appendix*, Table S1, A and B and Fig. S3*A*). Plug retention might also promote progression of the first male’s sperm although we were unable to detect significant evidence of this (*SI Appendix*, Table S1, A and B and Fig. S3*A*). Copulatory behavior differed when males mated in first or second mating roles, with longer copulation durations and more intromissions prior to ejaculation in the second mating role (*SI Appendix*, Table S2). However, neither variation in copulatory behavior (*SI Appendix*, Table S3) nor the interval between ejaculations (*n* = 17, *Χ*^2^ = 0.41, *P* > 0.50) predicted whether or not the first male’s plug was retained beyond the second male’s ejaculation.

**Table 1.** Influence of dislodgement or retention of the first male’s mating plug on the relative number of sperm from competing males that reach the uterus

Factor	_*1*_^2^	*P*
No. of second male’s sperm		
Retention of first male’s mating plug	5.42	0.02*
Total sperm no. in uterus after double mating	8.55	<0.01**

When two males mated with the same female, the relative number of their sperm varied significantly according to whether or not the first male’s mating plug was retained. The data are presented in Fig. 3*A*. Relatively fewer sperm from the second male were present in the uterus when the first male’s plug was retained. Results are shown for linear mixed models fit by maximum likelihood, testing the effect of plug dislodgement or retention on the number of sperm recovered from the second male for 16 double copulations in which sperm were recovered from both males, consisting of 16 males in role 2 (mating second) and 10 in role 1 (mating first). This excludes one case in which no sperm from the first male were recovered although this doesn’t significantly alter the outcome of the analysis. The identity of the first male to mate is included as a random factor. Bold text indicates statistical significance (*P < 0.05; **P < 0.01). χ^2 ^= Chi-squared.

Since it has been hypothesized that larger plugs are beneficial under sperm competition (8, 18), we also explored the consequences of natural variation in plug size. However, when testing for an association between plug size and sperm transport, it is important to recognize that larger ejaculates are likely both to contain more sperm and to result in the formation of larger plugs. To test for an influence of plug size on sperm transport, it is therefore important to take into account the total number of sperm ejaculated. Although it was not possible to quantify this directly, sperm counts from the cauda epididymis are a useful proxy. That is, the number of sperm available for ejaculation by a sexually rested male should predict the average number of sperm ejaculated under controlled conditions, notwithstanding that ejaculate traits can also be plastically adjusted to conditions at mating, such as sperm competition risk (19). Consistent with this expectation, cauda sperm counts predicted the number of ejaculated sperm recovered following a single copulation by subject males under controlled conditions (linear regression, *n* = 18, *r*^2^ = 0.45, F = 6.24, *P* = 0.01; cauda sperm count, *t* = 2.67, *P* = 0.02; body mass, *t* = 1.89, *P* = 0.08). To assess the potential role of the mating plug in promoting sperm transport into the uterus, cauda sperm counts were therefore included as a covariate in the analysis, together with body mass. Taking these variables into account, plug mass explained significant variation in the number of sperm recovered from the uterus immediately after ejaculation when subjects mated in both first (as a single mating) and second mating roles (Table 2 and *SI Appendix*, Fig. S3*B*). This analysis also reveals a significant influence of mating role on the number of sperm in the uterus, with more sperm recovered for males mating in the second role than the first (Table 2), although plug mass did not differ significantly according to mating role (paired *t* test, *n* = 14; *t* = 0.23, *P* > 0.80). Here, sperm numbers from the same subject males mating in first and second mating roles are directly comparable because each was collected immediately after ejaculation, unlike the comparison of first and second males mating sequentially with the same female, where there is a delay before recovery of the first male’s ejaculation. Hence, it appears that more sperm were ejaculated by focal males when mating in the second role, where sperm competition risk is elevated, and that the number of sperm ejaculated can vary independently of plug size. Larger plugs may therefore facilitate the transport of a greater proportion of ejaculated sperm to the uterus, with variation in the number of available sperm ejaculated linked to sperm competition risk (19).

**Table 2.** Influence of mating plug mass on the number of sperm from the plug-producing males that reach the uterus

Factor	*Χ*_*1*_^2^	*P*
Focal male sperm no. in uterus		
Mating role (first or second to mate)	4.10	<0.04*
Focal male mating plug mass (when only plug)	15.96	<0.001***
Cauda sperm count	2.56	0.11
Body mass	2.82	0.09

When subject males mated in either first (in single copulations) or second (in double copulations) mating roles, the number of their sperm recovered from the uterus immediately after ejaculation was predicted by the mass of their mating plug, as well as their mating role (first or second to mate). Body mass and cauda sperm count (measured postmortem as an index of sperm production) are included as covariates with the aim of controlling for variation in the number of sperm ejaculated (see *Results and Discussion*), which is likely to vary with plug mass. Results are shown for linear mixed models fit by maximum likelihood, for 18 males mating both first (in single copulations) and second (in double copulations) mating roles. Plug mass data exclude four cases for double copulations where proteomic analysis revealed that the plug originated from more than one male. Male identity is included as a random factor in the analysis. Bold text indicates statistical significance (*P < 0.05; ***P < 0.001).

Testing if larger mating plugs form a more effective barrier to rival ejaculates is more challenging because, in most cases, the first male’s plug is dislodged during sperm competition and lost, and so its mass cannot be determined directly (or if retained, it is mixed with plug material of the second male, and its mass cannot be determined accurately). However, plug mass for subjects in our study was strongly correlated with the mass of the seminal vesicles from which the major plug proteins originate (*SI Appendix*, Table S4 and Fig. S4*A*). Moreover, labeling of sperm proteins reveals that when the first male to mate with a female had relatively large seminal vesicles (and hence, by inference, was able to produce a large plug), fewer sperm from the second male were present in the uterus following a double copulation (A in Table 3 and Fig. 3*B*). By contrast, when the second male to mate had relatively large seminal vesicles, more sperm from the second male were present in the uterus following a double copulation (B in Table 3 and *SI Appendix*, Fig. S4*B*). These results are thus consistent both with the hypothesis that larger mating plugs are more efficient in blocking rival sperm and with the hypothesis that larger plugs are more effective in promoting transport of the subject male’s own sperm.

**Table 3.** Seminal vesicle masses of competing males predict the number of sperm in competition

Factor	Estimate ± SE	*t*	*P*
A) Sperm no. in uterus from second male			
Intercept	116.03 ± 32.15	3.61	
Seminal vesicle mass of first male	−0.23 ± 0.07	−3.22	<0.01**
Cauda sperm count of second male	0.28 ± 0.10	2.79	0.02*
B) Sperm no. in uterus from second male			
Intercept	−12.32 ± 28.27	−0.44	
Seminal vesicle mass of second male	0.19 ± 0.08	2.48	0.02*
Cauda sperm count of second male	0.32 ± 0.10	3.21	<0.01**

When two males mate with the same female, the number of sperm from the second male recovered from the uterus: A) decreases with seminal vesicle mass of the first male, and B) increases with seminal vesicle mass of the second male. Cauda sperm count of the second male (measured postmortem as an index of sperm production) is included as a covariate with the aim of controlling for variation in the number of sperm ejaculated (see *Results and Discussion*). Results are shown for linear mixed models fit by restricted maximum likelihood (REML), for 18 double copulations with 18 males mating second and 11 males mating first. The identity of the first male to mate is included as a random factor in the analysis. Bold text indicates statistical significance (*P < 0.05; **P < 0.01). *t *= t value.

Since our findings provide evidence that natural variation in both the retention and size of bank vole mating plugs influences the relative proportion of rival males’ sperm in competition, it is important to quantify how this affects reproductive fitness. Hence, a further series of double copulations was conducted to quantify paternity outcomes when the same subject males compete for fertilizations. Paternity outcomes, presented as P2 (the proportion of offspring in a litter sired by the second male to mate), were correlated with the relative proportion of rival males’ sperm in competition, as quantified by stable isotope labeling when the same male pairs mated in our first experiment (linear regression, *r*^2^ = 0.56, F_1,7_ = 8.9, *P* = 0.02) (*SI Appendix*, Fig. S5). We therefore conclude that, by influencing relative sperm numbers in competition, mating plugs function to increase male fertilization success under sexual selection.

These findings resolve a longstanding debate concerning the function of rodent mating plugs by revealing how they promote male fertilization success under sexual selection. Specifically, we show that the mating plugs of male bank voles can inhibit rival male ejaculates and also appear to promote sperm transport, each with important fitness consequences under sperm competition. The discovery of “double plugs” within the female reproductive tract demonstrates conclusively that plug material deposited by a female’s first mate can survive a second male’s copulation although, more commonly, the first male’s plug is completely dislodged. Since rodent plugs typically adhere tightly to the vaginal wall (6, 17), adjustments to the second male’s copulatory behavior may assist with plug dislodgement, and a similar pattern of increased copulatory effort by a female’s second mate has been reported in other rodent species (13, 20). However, we find no evidence that prolonged retention of the first male’s plug is a result of reduced dislodgement effort by the second male or of a delay in initiating the second copulation, as predicted if plugs become more difficult to remove with time since deposition (6). Variation in plug retention may therefore be explained by differences in how tightly the plug adheres to the vaginal epithelium, potentially resulting from the plug’s size or biochemical properties (8, 21), its position (22), and/or female-related factors (7, 17). For example, it has been hypothesized that female-derived endopeptidases might function in degradation of the mating plug, facilitating its detachment from female tissue as an initial step in dislodgement, and that endopeptidase inhibitors in male seminal fluid may partly function to protect the plug from such degradation (17).

Our finding that the rodent mating plug can inhibit rival males’ ejaculates is consistent with a hypothesized mechanical barrier mechanism under sperm competition (6). Moreover, the partial effectiveness of the in situ mating plug as a mechanical barrier provides empirical support for theoretical models predicting that plug efficacy should be lower when the probability of female remating is high (23, 24). To block a rival, the first male’s plug could physically obstruct the second male’s ejaculate from reaching the site of fertilization (2, 6). Alternatively or additionally, the presence of the first male’s plug might influence the stimulation experienced by the mating pair during the second male’s copulation, potentially resulting in the ejaculation and/or transport of fewer sperm.

Based on comparative evidence for rodents, it has previously been hypothesized that larger seminal vesicles and mating plugs are beneficial under sperm competition (8). Our study supports this hypothesis. Plug mass was strongly correlated with the mass of the seminal vesicles for subjects in our study, and, when the first male to mate had relatively large seminal vesicles, relatively fewer of the second male’s sperm were recovered from the female’s uterus. Larger plugs thus appear to form a more effective mechanical barrier. Plug and seminal vesicle mass also predicted the number of a male’s own sperm recovered from the uterus, as expected if larger plugs facilitate increased sperm transport. Although the evidence for an offensive function of the mating plug in our study is less direct than for a defensive function, our results complement findings of previous experimental studies in which ablation of seminal vesicles, gene knock-outs, or depletion of seminal fluid was used to impair rodent plug formation (10, 12, 13, 25–27). Such studies show that a diminished or absent mating plug leads to reduced sperm numbers in the uterus and oviducts (25–27) and reduced paternity success under competitive conditions (10, 13). These competitive benefits associated with the production of larger mating plugs, from both defensive and offensive perspectives, offer insight as to why, in a previous study, male bank voles developed larger seminal vesicles under social conditions linked to high sperm competition risk (18). However, there is also some evidence to suggest that smaller plugs may be more difficult for females to dislodge in the absence of further copulations (28).

Despite apparent benefits of larger plugs under sperm competition, we found no evidence of facultative adjustment in plug size linked to mating role. Similarly, two previous studies found no evidence that males adjust plug size according to sperm competition risk (29, 30), suggesting that adaptive variation in plug size may be subject to constraint in male rodents. By contrast, adaptive sperm allocation is both predicted by theory and reported under a range of competitive conditions (19, 31). Here, by labeling ejaculates of competing males, we were able to extend this evidence to demonstrate increased sperm allocation by male bank voles when ejaculating with a female that has recently mated.

In conclusion, our application of stable isotopes to label competing ejaculates demonstrates that longer retained and larger rodent mating plugs are beneficial under sperm competition. Males producing plugs that are retained for longer, and/or larger plugs, are able to increase the number of their sperm progressing within the female reproductive tract relative to those of a competitor following female multiple mating, ultimately resulting in more fertilizations. With appropriate labeling protocols, our approach is readily transplanted to other species. This application of proteomics to label competing ejaculates therefore opens new opportunities for investigation of postcopulatory processes among diverse animal taxa.

## Materials and Methods

### Subjects.

Subjects were from a captive colony, outbred for two or three generations and derived from local populations in Cheshire, United Kingdom. All males were individually housed in polypropylene cages (M3, 48 × 15 × 13 cm; North Kent Plastic Cages Ltd.) from age 35 d. Females were housed in sibling groups in polypropylene cages (MB1, 45 × 28 × 13 cm; North Kent Plastic Cages). Females were primed prior to mating by exposure to soiled substrate from the cages of random unrelated males. All animals were maintained under controlled environmental conditions, with temperature 21 °C ± 1 °C, relative humidity 45 to 65%, and a reversed light cycle (lights off 0830 to 1630). They were provided with ad libitum access to water and food (Lab Diet 5002 Certified Rodent Diet; Purina Mills, St. Louis, MO), with cage enrichment, substrate (Corn Cob Absorb 10/14), and paper wool nest material.

To distinguish the ejaculates of competing males following sequential copulations with the same female, males (*n* = 24) were fed a manipulated diet from age 2 mo. They were first acclimated to a reconstituted semisynthetic diet (prepared in-house), based on the 5002 Certified Rodent Diet supplemented with [^12^C_6_]lysine at a level equal to the natural lysine content of the diet (1.18% [wt/wt]), for 7 d prior to the start of the experiment. For “heavy” labeled males, this “light diet” was then replaced with a semisynthetic diet, identical apart from the substitution of the unlabeled crystalline amino acid by crystalline [^13^C_6_]lysine at the same level, yielding a relative isotope abundance (RIA) of 0.5. To create semisynthetic diets, the dietary pellets were dissociated with water containing the dissolved [^13^C_6_]lysine, to form a thick paste, and mixed extensively. When fully homogeneous, the paste was then extruded into strips ∼1 cm across and dried in a commercial foodstuff drying oven at 40 °C. This is referred to as “heavy diet.” Subjects then consumed the heavy (H, *n* = 8) or light (L, *n* = 16) diet for at least 40 d, to ensure that both seminal fluid proteins and sperm were fully labeled prior to mating (14). Subjects were randomly allocated to diet types, and fewer “heavy” subjects were produced than “light” in order to optimize the use of labeled diet. One additional “heavy” male and four “light” males were initially tested but not included in the final set of 24 males, due to inconsistent mating success during the experimental phase or problems with processing samples during the proteomic analysis phase. Feeding continued until experimental copulations were completed. Diet trials conducted prior to the study confirmed that reconstituted food pellets, and pellets with added lysine, did not differ in palatability, uptake of this diet was indistinguishable from normal diet, and growth curves were unchanged (32). Analyses conducted in the present study also confirmed that heavy (H) and light (L) labeled males did not differ significantly in their mean ejaculated sperm counts (mean ± SE sperm per microliter: H = 208,863 ± 37,155, L = 180,642 ± 17,531, *t*_18_ = 0.71, *P* = 0.49) or mating plug masses (mean ± SE plug mass (g): H = 0.0291 ± 0.007, L = 0.0270 ± 0.002, *t*_18_ = 0.42, *P* = 0.68) recorded following noncompetitive copulations.

### Quantifying Competing Ejaculates.

#### Double and single copulations.

To investigate the consequences of natural variation in mating plug characteristics on sperm numbers recovered from the uterus, a series of double and single copulations were conducted using differentially labeled males (*SI Appendix*, Fig. S6). Females from the stock population were mated with either two or one unrelated males (defined as having no shared parents). Twenty males (4H, 16L) mated second in a double copulation (second mating role) and alone (single copulation), and 12 (8H, 4L) mated first in a double copulation (first mating role) (*SI Appendix*, Fig. S6, and see below). This resulted in a total of 60 copulations (24H, 36L ejaculations by 8H and 16L males) with 40 females, 20 of which mated sequentially with two males (4LH and 16HL) and 20 of which mated with a single male (4H and 16L). Males were rested for at least 7 d after each ejaculation to prevent sperm depletion, and some were used more than once (range, one to four times) to mate first in double copulation trials.

Copulations took place in enclosures (70 × 60 × 60 cm) under red light with remote monitoring. Mating behavior was recorded to quantify copulation latency, copulation duration, and number of intromissions per copulation, as well as the interval between successive ejaculations in double copulations. Following introduction, pairs were initially monitored remotely for at least 20 min. If copulation commenced, remote monitoring and recording of behavior continued until ejaculation, or up to a maximum duration of 2 h, before ending the session. If no intromissions occurred within 20 m, the female was replaced, with a maximum of three females trialed per day for any male. For double copulations, first males were removed immediately after ejaculation, and second males were immediately introduced to the mated female. Behavior was recorded remotely as before until the second male ejaculated, or for up to 2 h. Mated females were removed from the experiment if they failed to remate (*n* = 5) or if the second male failed to ejaculate within 2 h of initiating a copulation (*n* = 1).

#### Ejaculate recovery and sample storage.

Immediately after each single or double copulation, female voles were humanely killed to recover ejaculates and mating plugs. Mating plugs were removed from the vagina, weighed, and stored at −20 °C for proteomic analysis. An incision was made along the entire length of each uterine horn to release the contents; the uterus and contents were then transferred to a 1.5-mL microcentrifuge tube with 200 µL of phosphate-buffered saline (PBS) solution and agitated in a vortex mixer on the lowest setting for 15 s to wash the contents of the uterus into solution. The PBS–ejaculate solution was then transferred to a clean 1.5-mL microcentrifuge tube. A further three washes were performed by adding 100 µL of PBS to the uterus, agitating each time using a vortex mixer on the lowest setting for 15 s, and adding the PBS–ejaculate solution from each wash to the previously recovered PBS–ejaculate solution. Hence, each uterus was washed four times in total, once in 200 µL of PBS solution and three times in 100 µL, with the wash solutions pooled for analysis. A sperm count was performed by diluting 10 µL of the uterus wash solution into 300 µL of 1% citrate solution. The number of sperm in the ejaculate was determined using a hemocytometer, and remaining samples were stored at −20 °C for proteomic analysis.

#### Proteomic analysis.

Proteomic analysis was conducted blind to other data collection. Mating plugs (n = 17) and ejaculates (*n* = 20) were analyzed to quantify relative contributions of competing males following 20 double copulations. Ejaculates (*n* = 18) from 20 single copulations were also analyzed to confirm the labeling status of subjects. That is, because the heavy labeled males were administered [^13^C_6_]lysine at an RIA of 0.5, it was important to ensure that the ejaculate proteins were fully labeled: i.e., had achieved an RIA for individual peptides of 0.5. This was assessed by measurement of the RIA of a tryptic peptide from the rapidly secreted seminal vesicle protein SVS4 (SASGSSTSYSLDK), which confirmed that all males had attained full labeling (*SI Appendix*, Fig. S7). The number of plugs analyzed for double copulations (*n* = 17) and number of ejaculates analyzed for single copulations (*n* = 18) were fewer than the maximum number of 20 each because samples were lost in processing.

Of the plugs recovered after double copulations and analyzed for protein content, most (12 of 17) were solid (did not split) on recovery and were cut into four to analyze the outer two quartiles (*SI Appendix*, Fig. S2). A smaller number (3 of 17) split naturally into two similar-sized sections on recovery, resembling two discrete plugs, and, in these cases, each part was cut into two for analysis (*SI Appendix*, Fig. S2). Two additional plugs were also cut into two for analysis. The first had a tiny portion that had broken off naturally but was too small to analyze separately, and the second was a complete plug that was relatively small on recovery (*SI Appendix*, Fig. S2).

Samples for proteomics were prepared using standard protocols (32). In brief, samples were solubilized in a mass spectrometry compatible detergent (Rapigest; Waters), reduced, and alkylated, prior to digestion with trypsin. Tryptic peptides were analyzed using an UltiMate 3000 RSLCnano System (Thermo Scientific, Hemel Hempstead) coupled to a QExactive mass spectrometer (Thermo Scientific). Samples were loaded onto the trapping column (PepMap100, C18, 300 μm × 5 mm; Thermo Scientific), using partial loop injection, for 7 min at a flow rate of 4 μL⋅min^−1^ with 0.1% (vol/vol) formic acid and resolved on the analytical column (Easy-Spray C18, 75 µm × 500 mm 2-µm column) using a gradient of 97% A (0.1% formic acid) 3% B (99.9% acetonitrile, 0.1% formic acid) to 60% A 40% B over 15 min at a flow rate of 300 nL⋅min^−1^. The data-dependent program used for data acquisition consisted of a 60,000-resolution full-scan mass spectrometry (MS) scan (automatic gain control set to 3e6 ions with a maximum fill time of 100 ms); the seven most abundant peaks were selected for tandem mass spectrometry (MS/MS) using a 60,000-resolution scan (AGC set to 1e5 ions with a maximum fill time of 110 ms) with an ion selection window of 1.2 *m*/*z* and a normalized collision energy of 30. To avoid repeated selection of peptides for MS/MS the program used a 10-s dynamic exclusion window. The raw liquid chromatography (LC)-MS files were uploaded onto Proteome Discoverer (version 1.4.1.14; Thermo Scientific) software and searched against an in-house–generated protein sequence database produced from running National Center for Biotechnology Information (NCBI) “blastp” searches of individual translated sequences obtained from an *M. glareolus* whole genome sequence against all UniProt entries for “Chordata” (33). The top hit (that with the lowest e-value and highest bit score) for each sequence was used to create a protein sequence database. In addition, peptides sequenced de novo from seminal vesicle-secreted proteins were added to the database. The final protein database used here is included as Dataset S1. Search parameters included carbamidomethylation of cysteine as a fixed modification, and both methionine oxidation and [^13^C_6_]lysine as variable modifications. To determine heavy:light ratios, the raw files were loaded onto Skyline (version 4.1). For mating plugs, the extracted ion chromatograms (XICs) of the monoisotopic ion of the heavy (*m*/*z* of 648.306; z = 2) and the monoisotopic ion of the light (*m*/*z* of 645.296; z = 2) SVS4 peptide SASGSSTSYSLDK were used to determine the area under the curve.

Peptides originating from “heavy” and “light” males have different mass; light males contribute only light peptides while heavy males contribute both heavy and light peptides in equal proportion. Thus, if the proportional abundances of the monoisotopic ion of the heavy and light forms of a peptide are *A*_*H*_ and *A*_*L*_ respectively (expressed as a fraction of the total), the peptide abundance contributed by the heavy male is equal to *2A*_*H*_ and from the light male *A*_*L*_ *− A*_*H*_ (Fig. 1). The heavy:light ratio of ejaculate-derived peptides can therefore be used to determine the proportional abundance of sperm and plug-derived proteins originating from each male following a double mating.

#### Quantifying relative sperm numbers from competing males.

Data on the proportional abundance of sperm-derived proteins were used to quantify the relative number of sperm recovered from competing males following double copulations. Initially, data were obtained from a total of 165 peptides derived from 46 proteins (*SI Appendix*, Table S5). However, since not all of these were sperm derived, we performed a series of steps in order to identify those peptides that could be reliably used to calculate sperm numbers for each male. First, by analyzing four ejaculates recovered from females that had mated once with a heavy labeled male, we were able to identify and eliminate proteins that were unlikely to be male-specific. Proteins were excluded if less than 90% of the protein abundance was attributable to the heavy male. Secondly, we tested if the abundance of each candidate protein was significantly correlated with total sperm count within each sample so that proteins that did not correlate with ejaculate sperm counts could be removed from the calculation. Relationships of protein abundance with sperm counts were tested using ordinary least squares regression models, with an alpha level of 0.05 adjusted by Bonferroni correction to 0.002 to account for the testing of 46 proteins. These filters led to exclusion of 22 proteins (20 that were unlikely to be male-specific and 2 that did not correlate significantly with ejaculate sperm counts). Finally, we inspected the remaining 24 proteins to confirm that they were likely to originate from sperm (e.g., sperm structural or mitochondrial proteins). Three further proteins were excluded, leaving 21 proteins yielding 96 lysine-containing peptides. Since these remaining 96 peptides originated from sperm, the heavy:light ratio for each peptide should be constant within each double copulation. For example, if the heavy male contributed twice as many sperm as the light male, double the abundance of each sperm-derived peptide would also be attributable to the heavy male. We therefore flagged peptides where the heavy:light ratio differed significantly from other peptides in the same ejaculate, setting an outlier threshold of a heavy:light ratio greater than 2 interquartile ranges (IQRs) above the upper quartile or less than 2 IQRs below the lower quartile for each individual ejaculate. Peptides were excluded if they were outliers in five or more samples, leading to exclusion of 13 peptides. These variances are probably due to weak precursor ion signals that are prone to contamination by other ions. At the end of this stringent filtration, 83 peptides, derived from 19 different proteins, remained (*SI Appendix*, Table S6). The median heavy:light ratio of these 83 peptides for each sample was used to define the proportion of sperm that originated from the heavy and light males, respectively (Fig. 1). These proportions were then applied to the total sperm count in the ejaculate, giving the total number of sperm contributed by each male.

### Paternity Success under Sperm Competition.

To quantify paternity outcomes, a further series of double copulations were conducted ∼12 wk after the first double copulation experiment. This was to test if relative sperm numbers of competing males in the uterus following a double copulation are predictive of paternity outcomes. Our approach therefore relies on the outcome of controlled double copulations, with the same two males being broadly repeatable, although there is potential for variability: for example, due to within-male variation in ejaculate composition or female effects (19). Potential sources of within-male variation were therefore minimized as far as possible by replicating the same carefully controlled environmental conditions, with males rested to prevent sperm depletion and each allowed only a single ejaculation. Under such conditions, ejaculated sperm numbers and the size of copulatory plugs should be largely determined by daily sperm production rates and the size of the sexual accessory glands, respectively (8, 34), which were unlikely to have altered significantly over the relatively short timescale of the experiments. Similarly, although less is known about factors determining plug retention, we are assuming this will be influenced by repeatable biochemical properties of the plug or ejaculate. A degree of repeatability in relative sperm numbers reaching the uterus is therefore expected when the same males compete under controlled conditions, notwithstanding potential female-mediated effects. Nine females successfully weaned a litter following a double copulation with one of the same male pair combinations used in the first double copulation experiment, for which data were also available on relative sperm numbers. After receiving a single ejaculate from both the first and second male, as described above, females were individually housed in standard laboratory cages to rear litters. Tissue samples (5-mm ear punch taken postmortem, stored at −20 °C) from parents and offspring were used to assign paternity with microsatellite markers.

Paternity analysis was conducted blind to findings of the initial double copulation experiment. DNA was extracted using a QIAGEN DNeasy Blood & Tissue Kit (QIAGEN, West Sussex, United Kingdom). Haplotypes were established by genotyping parents and offspring using up to six microsatellite markers (selected from ref. 35) (*SI Appendix*, Table S7). The forward primer for each marker was 5′-fluorescently labeled with 6-FAM, PET, or VIC. The loci were organized into two multiplex loading groups, containing mixed loci from three regions. PCR amplification reactions were performed in a 10-μL volume of 20 ng of DNA, 0.25 to 0.5 μM primer, and 5 μL of BioMix Red reaction mix (Bioline, London, United Kingdom). The PCR protocol steps were as follows: an initial denaturation for 2 min at 94 °C; 35 cycles of denaturation at 95 °C for 45 s, annealing at 55 °C for 45 s, and extension at 72 °C for 1 min, and, after the 30 cycles were complete, a final extension at 72 °C for 5 min. The PCR reactions were then diluted to 16- to 25-fold (depending on primer set) and multiplexed in formamide with GeneScan LIZ500 size standard (Applied Biosystems). Haplotype size was determined with an ABI PRISM 3100 DNA analyzer and GeneMapper v3.0 software (Applied Biosystems).

Paternity outcomes were quantified unambiguously as P2, the proportion of offspring sired by the female’s second mate, for comparison with relative sperm numbers, S2, the proportion of second male’s sperm recovered from the uterus following a double copulation in the previous experiment.

### Male Reproductive Morphology.

At least 1 wk after the last mating, male subjects were humanely killed to recover reproductive organs. Body mass and seminal vesicles mass (combined) were recorded, and an epididymal sperm count was performed. The cauda epididymis was macerated with a scalpel blade in 200 µL of PBS and left to stand for 2 min, before removing the sperm–PBS solution. Ten microliters of the sperm–PBS solution was diluted in 1 mL of 1% citrate solution, and sperm concentration was determined using a hemocytometer. Postmortem data could not be collected for two subjects.

### Statistical Analysis.

Statistical analyses were performed in R (3.4.0) (36). Linear mixed models (LMMs) were performed in R using the package lme4 (37), with subject identity as a random factor where individuals were used more than once in mating experiments (further details for each analysis are provided in table legends). Labeling status (heavy or light) was not included as an additional random factor in the analyses presented as this had no detectable influence on variables of interest in initial tests. Residuals and quantile-quantile-plots of all LMMs were visually inspected, and the distributions of residuals were compared to a normal distribution using Shapiro–Wilk tests. If residuals were not normally distributed, a square root or log transformation was applied, and residuals were rechecked. To obtain *P* values of LMM fixed effects, we used the mixed() function in the package “afex” (38) with a likelihood ratio (LRT) method. A linear regression analysis was performed with ArcsinSqrt transformed data for proportions (proportion of offspring sired [P2] and proportion of sperm recovered [S2] from the second male in double copulations).

### Data Accessibility.

This publication is supported by multiple datasets. The mass spectrometry proteomics data have been deposited to the ProteomeXchange Consortium via the PRIDE (39) partner repository with the dataset identifier PXD011694. All other data are provided in *SI Appendix*.

### Ethical Statement.

All animal care and experimental protocols were in accordance with the University of Liverpool Animal Welfare Committee requirements, EU Directive 2010/63/EU, and the UK Home Office code of practice for the housing and care of animals bred, supplied, or used for scientific purposes. The University of Liverpool Animal Welfare Committee approved the work, but, as stable isotope labels could be fed noninvasively to animals through their normal diet, no specific licenses were required to carry out the work.

## Supplementary Material

Supplementary File

Supplementary File

Supplementary File

Supplementary File

## Data Availability

Mass spectrometry proteomics data have been deposited in the ProteomeXchange Consortium via the PRIDE partner repository (dataset identifier PXD011694). All other study data are included in the article, *SI Appendix*, and Datasets S1–S3.
