# Supplementary material for: Revealing mechanisms of mating plug function under sexual selection
Source: Proc Natl Acad Sci U S A. 2020 Oct 19;117(44):27465–73. doi: 10.1073/pnas.1920526117 (PMC7959524; doi:10.1073/pnas.1920526117)
Supplement: Supplementary File [file pnas.1920526117.sapp.pdf]

Supplementary Information

Revealing mechanisms of mating plug function under sexual selection

Paula Stockley, Catarina Franco, Amy J. Claydon, Amanda Davidson, Dean E. Hammond,

Philip J. Brownridge, Jane L. Hurst, Robert J. Beynon

Supplementary figure legends

Fig. S1. Comparison of SVS4 peptides with other peptide data. The copulatory plug is an intractable material to deal with, as it is extensively cross-linked. It contains multiple proteins derived from the secretory vesicle. However, in terms of extractable proteins (SVS2 is the protein that contains the repetitive cross-linked sequence, and is not extractable), the most abundant protein was SVS4. When extracted from the plug, this protein appears on reducing SDS-PAGE as a polymeric ladder, implying cross-linking of 1,2 3.. copies of the protein. Label-free quantification of the extractable plug proteome revealed that SVS4 was two orders of magnitude more abundant that other proteins. This protein consistently yielded a high-quality peptide from which we were able to recover a clean extracted ion chromatogram and mass spectrum, and allowed us to assess whether the copulatory plugs were derived from the unlabelled male, the labelled male, or both. Where other peptides could be obtained, we analysed the labelling pattern. Orange symbols: other SVS4 peptides, blue symbols: peptides from other proteins (see the accompanying table). In every instance, the categorisation of the plugs according to donor male was unchanged, irrespective of the protein/peptide used (these are individual values, obtained from portions of the plugs, and need to be interpreted in the context of Fig. S2).

www.pnas.org/cgi/doi/10.1073/pnas.1920526117

Fig. S2. Recovered mating plugs. Plugs were recovered from female bank voles immediately after sequential copulation with two males, during which each male ejaculated once. Images of these plugs are numbered 1 to 17. Males mating in first or second mating roles had been fed either 'heavy' (H) or 'light' (L) diets to facilitate discrimination of their ejaculates, with the 'heavy' diet containing a stable isotope labelled amino acid ([$^{13}C_6$] lysine) at a relative isotope abundance of 0.5. For each plug, the identity and labelling status of the first male to mate is presented first in square brackets, with H1-4 indicating the identity of a 'heavy' first male, and L1-L14 indicating the identity of a 'light' first male. The identity and labelling status of the second male is then presented, using the same abbreviations. Mating plugs were cut to analyse the outer two quartiles (1-4, 8,9, 12-17), or if plugs split into two naturally, each part was cut in half (6,7,11, labelled as parts 1 and 2). A further two plugs were also cut in half (5,10 – see main text). The ratio of H/(H+L) was calculated based on the area of a potential SVS4 tryptic peptide SASGSSTSYSLDK (see main text). In most cases (2-5, 8-10, 12-17), proteomic analysis revealed that the recovered plug material originated entirely from the second male to mate: when the second male was 'light' (coloured blue in the figure) the plug contained no [$^{13}C_6$] lysine (H/(H+L)<0.04), and when the second male was 'heavy' (coloured red in the figure) the plug contained half (50% [$^{13}C_6$] lysine (H/(H+L)>0.47), consistent with full labelling. In four cases (1,6,7,11) proteomic analysis revealed that recovered plug material originated from both the first and second males to mate (coloured purple in the figure). Plugs 6,7 and 11 each split naturally into two on recovery. In these cases the section of the plug positioned closest to the cervix (part 1) contained protein from the first (heavy) male combined with protein from the second (light) male, whereas the plug section positioned behind this (part 2) contained protein only from

the second (light) male. Plug 1 did not split naturally on recovery but contained protein from both the second (heavy) and first (light) male.

Fig. S3. Influence of mating plugs on the number of sperm from competing males reaching the uterus. A. Shows variation in the mean (+ s.e) absolute number of sperm from the first and second males reaching the uterus following a double copulation when the first male's plug was dislodged or retained. For statistical analysis with control for sperm numbers recovered when males mated alone, see Table S1. **B.** The mass of the mating plug that males produced explained significant variation in the number of their own sperm recovered from the uterus immediately after ejaculation. More sperm were recovered for males producing larger plugs, when mating in either a first mating role (as the only male) or second mating role (in a double mating). For statistical analysis, see Table 2.

Fig. S4. Mass of seminal vesicles predicts plug mass and sperm transport. A. The mass of males' seminal vesicles predicted the mass of their mating plugs when mating in either first or second mating roles. Data are presented for 18 males, excluding 4 plugs of mixed origin recovered after double copulations. For statistical analysis with control for male body mass, see Table S4. **B.** Following double copulations, the number of the second males' sperm reaching the uterus was predicted by the mass of their seminal vesicles, and hence by inference, by the size of their mating plug. Larger seminal vesicles of the second male were associated with more of their sperm reaching the uterus. For statistical analysis with control for second males' cauda sperm count (as measure of sperm production) see Table 3A.

Fig. S5. Sperm competition and paternity outcomes. Following double copulations, the relative proportion of sperm from two competing males in the uterus explained significant variation in paternity outcomes when the same males mated again with a different female. S2 is the proportion of sperm present in the uterus from the second male immediately after a double copulation, and P2 is the proportion of offspring sired by the second male in a subsequent double copulation. Linear regression analysis: $r^2 = 0.56$, $F_{1,7} = 8.9$, $P= 0.02$.

Fig. S6. Overview of methodological approach. Male bank voles were fed on a manipulated diet to facilitate discrimination of their ejaculates under sperm competition. 'Heavy' (H) males consumed a diet containing 50% [$^{13}C_6$] lysine, and 'light' (L) males consumed a control diet with [$^{12}C_6$] lysine for a minimum of 40 days prior to experimental mating. To investigate the consequences of natural variation in mating plug characteristics, a series of double and single copulations were conducted using differentially labelled males. A total of 60 copulations were achieved with 40 females, 20 of which mated with a single male (4H and 16L), and 20 of which mated sequentially with two differentially labelled males (16HL, 4LH). Immediately after each single or double copulation, female voles were humanely killed to recover ejaculates and mating plugs. Mating plugs were removed from the vagina, the contents of the uterus were removed, sperm counts were performed and the ejaculated seminal fluid and sperm were separated for subsequent proteomic analysis.

Fig. S7. Isotope labelling of peptides from mating plugs.

Ejaculates were collected from unlabelled or 'heavy' labelled males, following a single copulation with a previously unmated female, and analysed by proteomics. The raw data files were interrogated to display the mass spectrum of a single peptide (from seminal

vesicle protein IV (SVS4 peptide SASGSSTSYSLDK, m/z 645.296 light, m/z 648.306, heavy) to illustrate the differences in labelling profiles. Samples are from 4 'heavy' labelled males (H1-H4), and 14 'light' males (L1-L14). Note the absence of any peak corresponding to the heavy peptide in the light labelled samples (L1 to L14).

Supplementary Information Datasets

Supplementary Excel spreadsheet

Filename: Data files_mating plugs.xlsx

This multi-tab spreadsheet contains raw data used for each of the analyses. Tab 1 is the index relating Tabs 2-15 to the location of results for each dataset, with a description of the analyses used, including number of observations, dependent variables, factors and random effects in models.

Supplementary Excel spreadsheet

Filename: All plug proteome data.xlsx

This multi-tab spreadsheet is the output of Proteome Discoverer (Thermo). Tab 1 is the index relating individual sample IDs to proteomics file numbers. Tab 2 is the overall proteome abundance for the protein profile, heavily dominated by SVS4. Finally, a standard Proteome Discoverer analysis is present in tab 3.

Supplementary FASTA database

Filename: BankVoleProteinDB_fasta.txt

This database was constructed by reference to other rodent protein databases, as described in the text. Where proteins were manually searched and annotated, these are indicated by short accession numbers (e.g. ‘BV4’, the most abundant protein in plugs, equivalent to SVS4 in other rodents).

Figure S1.

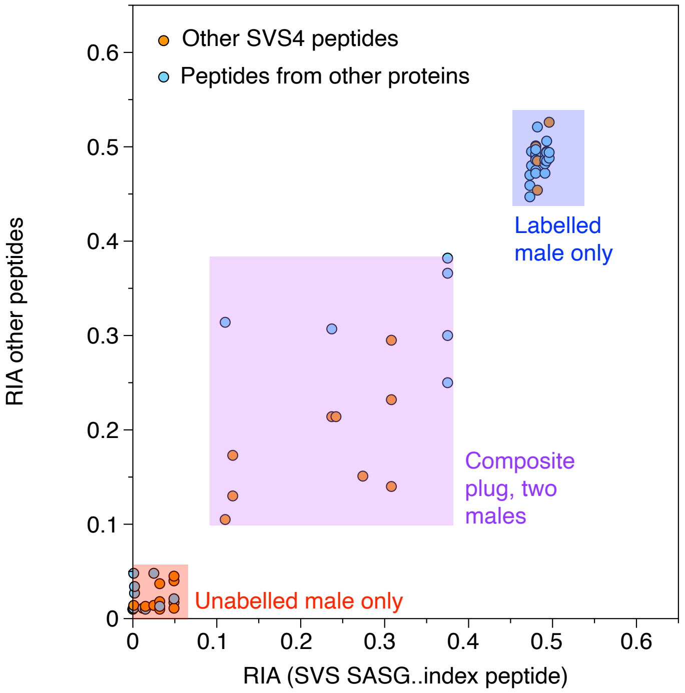

List of additional peptides used in Figure S1, with the calculated RIA values:

RIA: SVS4/SASG..index peptide	RIA: Other peptide	Protein/sequence	Sample ID	From SVS4?
0	.01	SVS4/FSQSEETSETYVTGSGEK	12366_4	Y
0	.01	SVS4/FSQSEESTSETYVTGSGEK	12349_1	Y
.001	.011	SERPIN/TQIMEGLGLSLQQSQEAELHK	12070_4	N
.001	.048	CAECAM10/VQVFSWYK	12696_4	N
.001	.014	SVS4/FSQSEESTSETYVTGSGEK	12696_4	Y
.002	.027	A1AT/QINDYVADETQGK	12111_1	N
.002	.034	AIAT/QINDYVADETQGK	12111_1	N
.012	.011	IL22R/VFIIDNLLEK	12111_2	N
.015	.01	SERPIN/DNFLSAMK	12070_1	N
.015	.013	SVS4/AVASSSEESYEESSHKK	12070_1	Y
.025	.048	CAECAM10/VQVFSWYK	12696_4	N
.025	.014	SVS4/FSQSEESTSETYVTGSGEK	12696_4	Y
.032	.018	SVS4/AVASSSEESYEESSHK	12261_1	Y
.032	.01	SVS4/AVASSSEESYEESSHKK	12261_1	Y
.032	.037	SVS4/FSQSEESTSETYVTGSGEK	12261_1	Y
.032	.013	SERPIN/TQIMEGLGLSLQQSQEAELHK	12261_1	N
.049	.017	SERPIN/MQQVESSLSEWALK	12030_1	N
.049	.011	SVS4/AVASSSEESYEESSHK	12030_1	Y
.049	.021	TIMPX1/NGNLHISACSFLPWHSLNAAQQK	12030_1	N
.049	.04	SVS4/AVASSSEESYEESSHKK	12030_1	Y
.049	.045	SVS4/FSQSEETSETYVTGSGEK	12030_1	Y
.11	.105	SVS4/AVASSSEESYEESSHKK	12361_2	Y
.110	.314	B2C/INVYYNEATGGK	12631_2	N
.119	.13	SVS4/AVASSSEESYEESSHK	12439_2	Y
.119	.173	SVS4/FSQSEESTSETYVTGSGEK	12439_2	Y
.237	.214	SVS4/AVASSSEESYEESSHKK	12361_1	Y
.237	.307	B2C/INVYYNEATGGK	12631_1	N
.274	.151	SVS4/AVASSSEESYEESSHK	12439_1	Y
.308	.232	SVS4/AVASSSEESYEESSHK	12364_4	Y
.308	.14	SVS4/AVASSSEESYEESSHKK	12364_4	Y
.308	.295	SVS4/AVASSSEESYEESSHKK	12364_4	Y
.375	.3	TGM/FSVESLALSNMQSWNQEK	12417_1	N

.375	.25	A1AT/EEELSSWVLVK	12417_1	N
.375	.382	SERPIN/MQQVESSLSEWALK	12417_1	N
.375	.366	TGM/FSVESLALSNMQSWNQEK	12417_1	N
.473	.447	CAECAM10/VQVFSWYK	12365_1	N
.473	.47	SERPIN/DNFLSAMK	12365_1	N
.473	.459	SERPIN/MQQVESSLSEWALK	12365_1	N
.475	.495	TIMPX1/FAYTPAQESLCGYVHK	12260_4	N
.475	.480	SERPIN/QINDYVAK	12260_4	N
.48	.501	SVS4/AVASSSEESYEESSHK	12260_1	Y
.48	.474	SERPIN/QINDYVAK	12260_1	N
.480	.500	SVS4/FSQSEESTSETYVTGSGEK	12260_1	Y
.480	.492	TIMPX1/FAYTPAQESLCGYVHK	12260_1	N
.480	.475	A1AT/EEELSSWVLVK	12260_1	N
.480	.488	A1AT/IIAVNFK	12260_1	N
.480	.472	AIAT/QINDYVADETQGK	12260_1	N
.480	.492	SERPIN/DNFLSAMK	12260_1	N
.480	.497	SERPIN/MQQVESSLSEWALK	12260_1	N
.480	.486	CAECAM10/SVVLLVHDLPEK	12260_1	N
.482	.485	SVS4/AVASSSEESYEESSHK	12364_1	Y
.482	.485	SVS4/AVASSSEESYEESSHK	12364_1	Y
.482	.454	SVS4/AVASSSEESYEESSHKK	12364_1	Y
.482	.521	SERPIN/TLYMADTFSTNFGNPEMAK	12364_1	N
.491	.472	CAECAM10/VQVFSWYK	12365_4	N
.491	.482	SERPIN/DNFLSAMK	12365_4	N
.491	.486	SERPIN/MQQVESSLSEWALK	12365_4	N
.491	.486	SERPIN/FSIEGTYHLEK	12365_4	N
.493	.506	TIMPX1/FAYTPAQESLCGYVHK	12693_4	N
.493	.485	CAECAM10/SVVLLVHDLPEK	12693_4	N
.493	.495	SERPIN/LSLYLPK	12693_4	N
.493	.494	SERPIN/DNFLSAMK	12693_4	N
.496	.488	TIMPX1/FAYTPAQESLCGYVHK	12693_1	N
.496	.494	CAECAM10/WFLNGK	12693_1	N
.496	.526	SVS4/FSQSEESTSETYVTGSGEK	12693_1	Y
.242	.214	SVS4/AVASSSEESYEESSHK	12417_1	Y

Figure S2

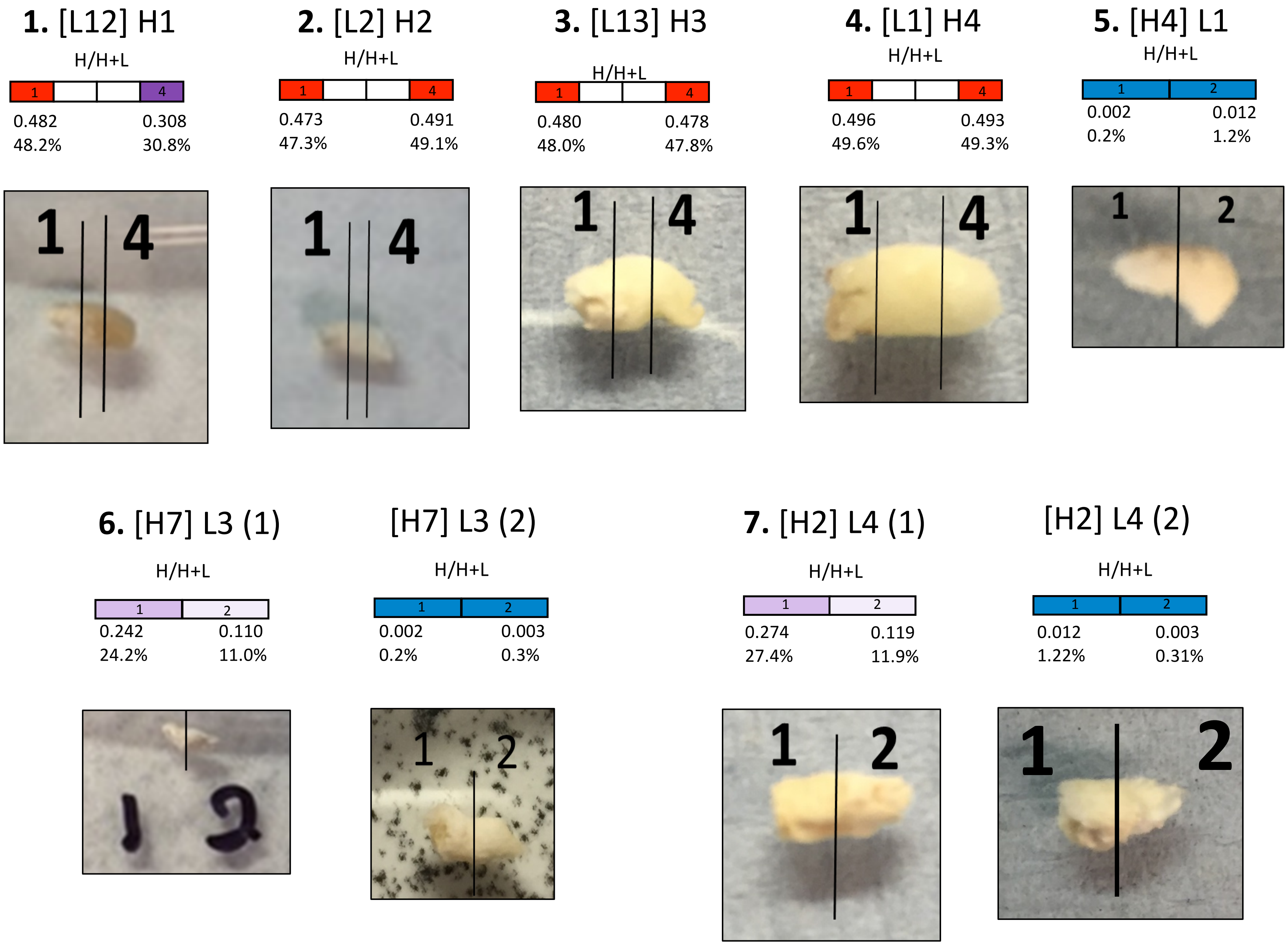
1. [L12] H1
H/H+L
1 4
0.482 48.2%
0.308 30.8%
1 4
2. [L2] H2
H/H+L
1 4
0.473 47.3%
0.491 49.1%
1 4
3. [L13] H3
H/H+L
1 4
0.480 48.0%
0.478 47.8%
1 4
4. [L1] H4
H/H+L
1 4
0.496 49.6%
0.493 49.3%
1 4
5. [H4] L1
H/H+L
1 2
0.002 0.2%
0.012 1.2%
1 2
6. [H7] L3 (1)
H/H+L
1 2
0.242 24.2%
0.110 11.0%
1 2
[H7] L3 (2)
H/H+L
1 2
0.002 0.2%
0.003 0.3%
1 2
7. [H2] L4 (1)
H/H+L
1 2
0.274 27.4%
0.119 11.9%
1 2
[H2] L4 (2)
H/H+L
1 2
0.012 1.22%
0.003 0.31%
1 2

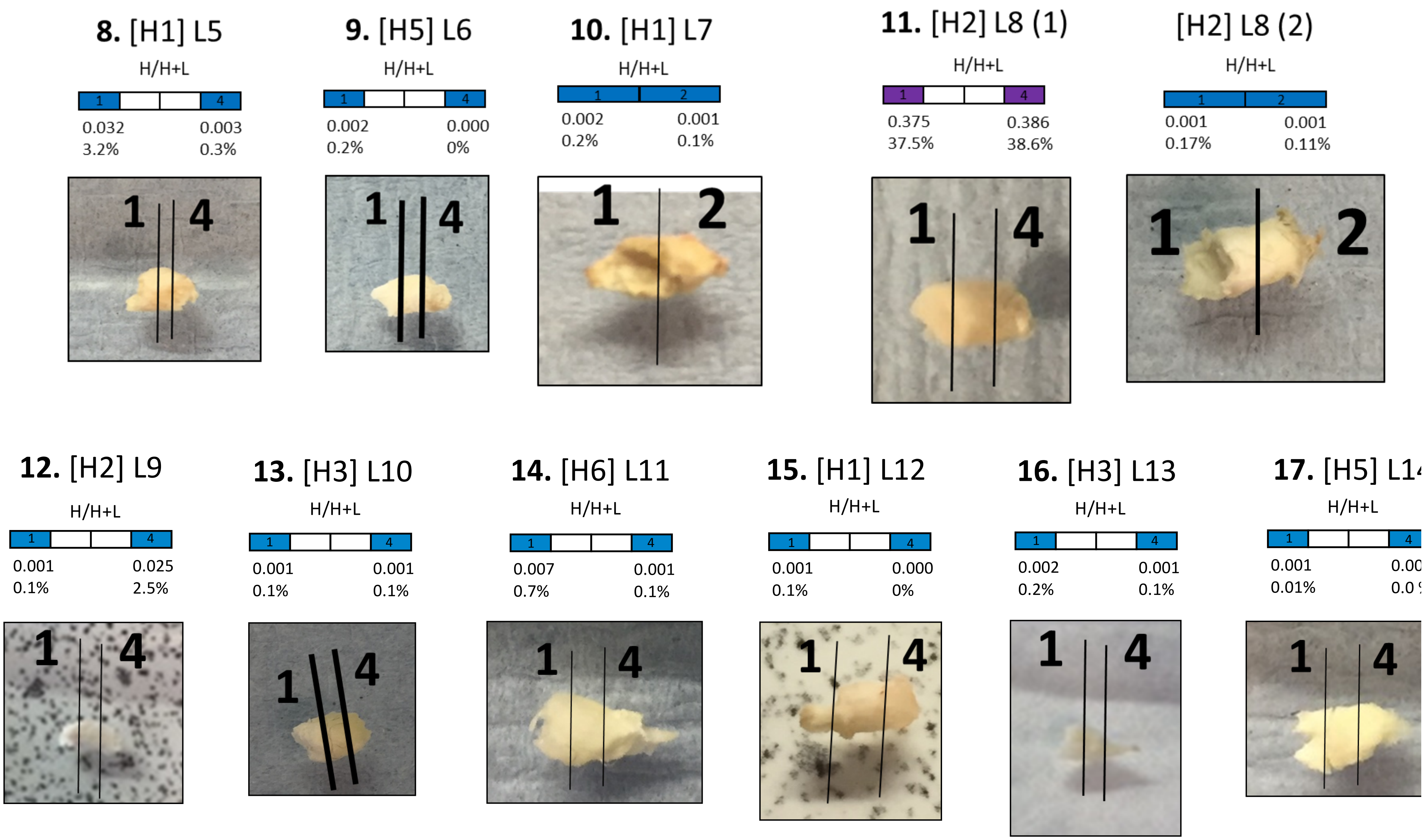
8. [H1] L5
H/H+L
0.032
3.2%
0.003
0.3%
9. [H5] L6
H/H+L
0.002
0.2%
0.000
0%
10. [H1] L7
H/H+L
0.002
0.2%
0.001
0.1%
11. [H2] L8 (1)
H/H+L
0.375
37.5%
0.386
38.6%
[H2] L8 (2)
H/H+L
0.001
0.17%
0.001
0.11%
12. [H2] L9
H/H+L
0.001
0.1%
0.025
2.5%
13. [H3] L10
H/H+L
0.001
0.1%
0.001
0.1%
14. [H6] L11
H/H+L
0.007
0.7%
0.001
0.1%
15. [H1] L12
H/H+L
0.001
0.1%
0.000
0%
16. [H3] L13
H/H+L
0.002
0.2%
0.001
0.1%
17. [H5] L1
H/H+L
0.001
0.01%

Figure S3

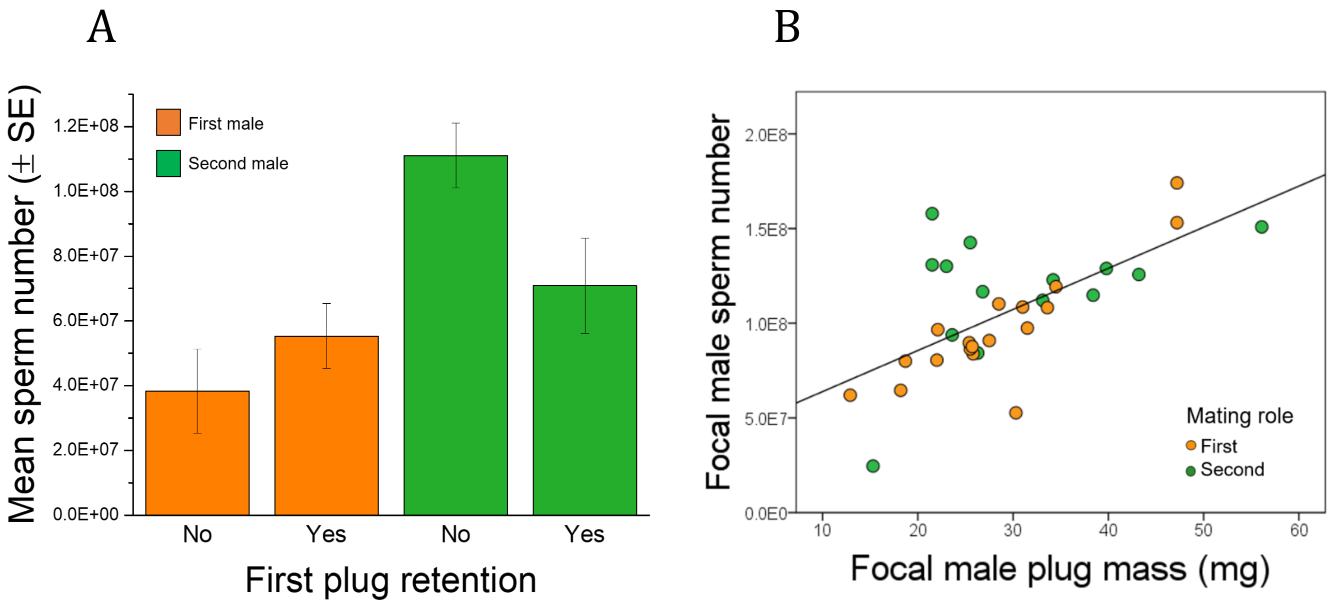

A
B
First male
Second male
Mean sperm number (± SE)
0.0E+00
2.0E+07
4.0E+07
6.0E+07
8.0E+07
1.0E+08
1.2E+08
No
Yes
No
Yes
First plug retention
Focal male sperm number
0.0E0
5.0E7
1.0E8
1.5E8
2.0E8
10
20
30
40
50
60
Focal male plug mass (mg)
Mating role
First
Second

Figure S4

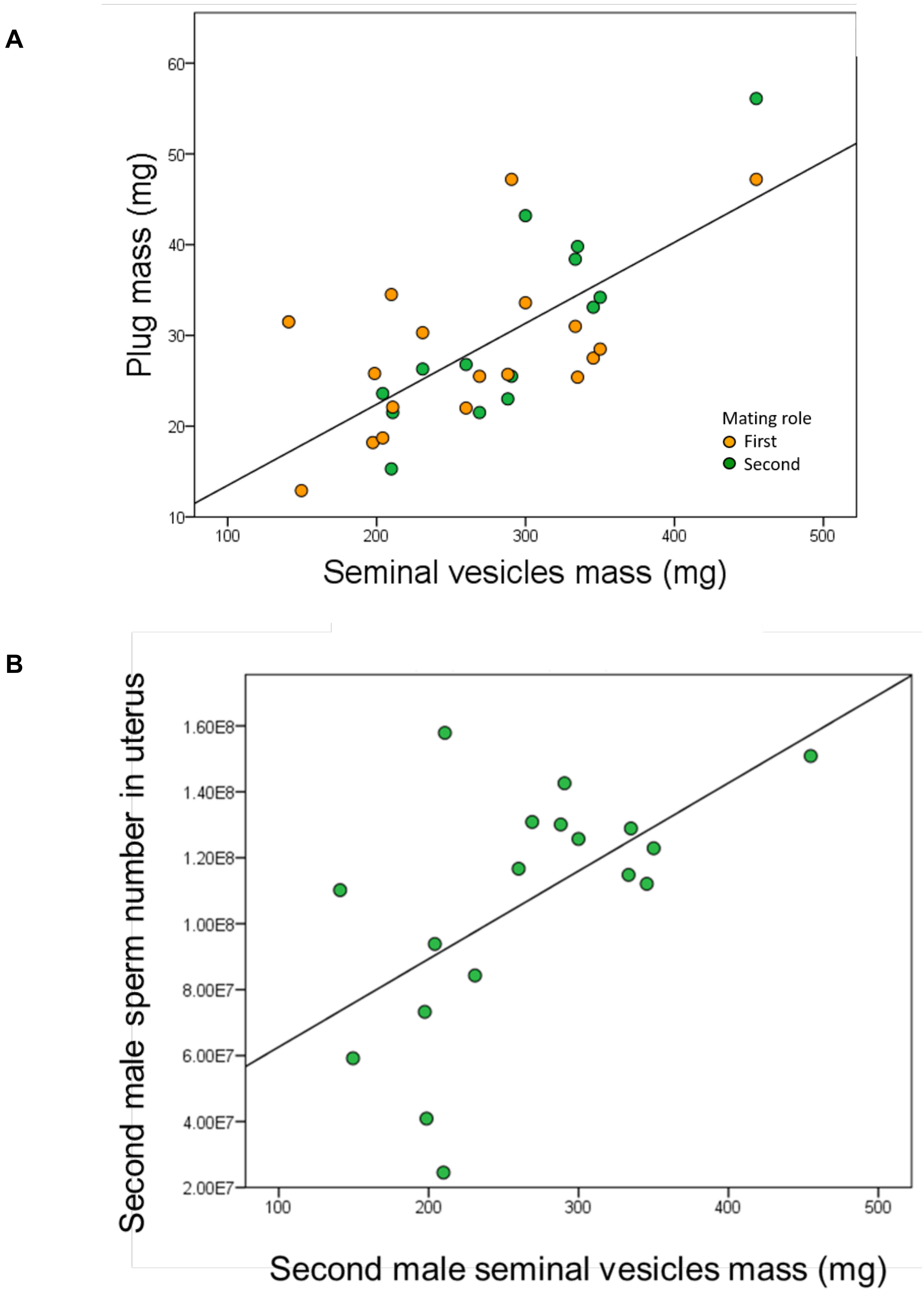
A
Plug mass (mg)
60
50
40
30
20
10
100
200
300
400
500
Seminal vesicles mass (mg)
Mating role
First
Second
B
Second male sperm number in uterus
1.60E8
1.40E8
1.20E8
1.00E8
8.00E7
6.00E7
4.00E7
2.00E7
100
200
300
400
500
Second male seminal vesicles mass (mg)

Figure S5

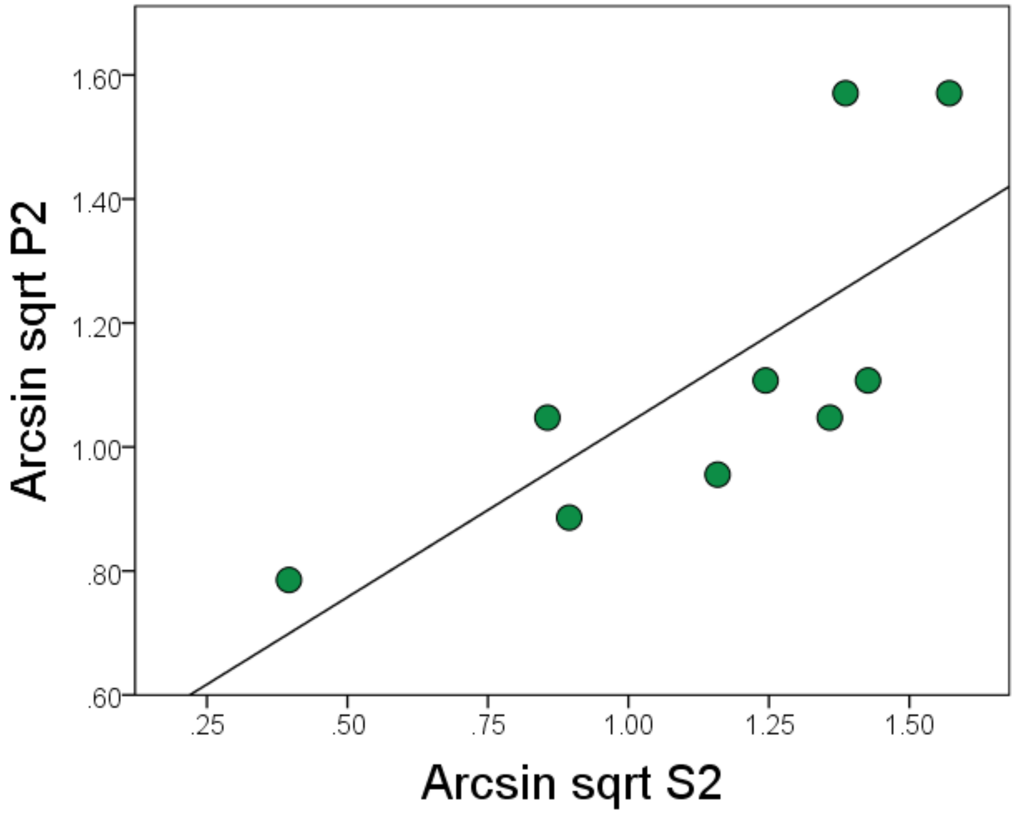
1.60
1.40
1.20
1.00
.80
.60
Arcsin sqrt P2
.25
.50
.75
1.00
1.25
1.50
Arcsin sqrt S2

Figure S6

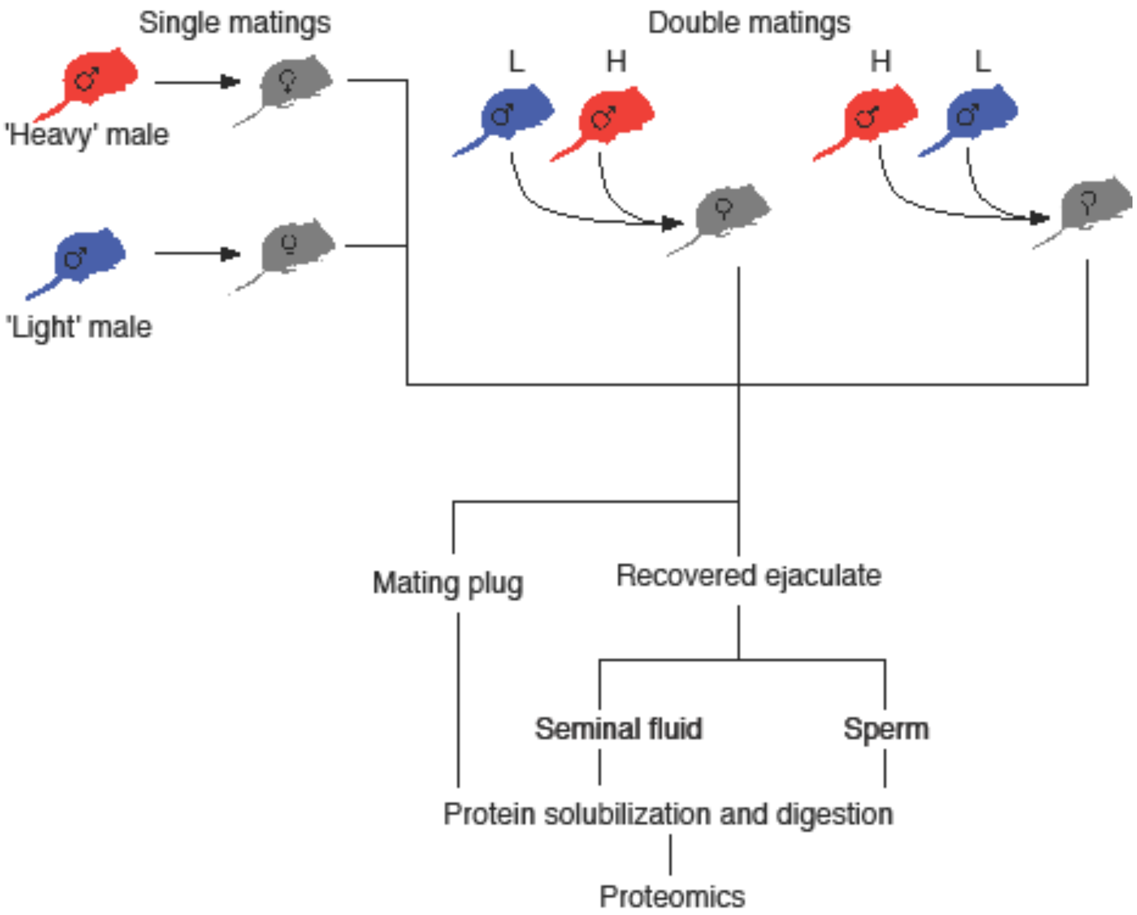
Single matings
Double matings
L
H
H
L
'Heavy' male
'Light' male
Mating plug
Recovered ejaculate
Seminal fluid
Sperm
Protein solubilization and digestion
Proteomics

Figure S7

A. SVS4 in ejaculates recovered following single copulations by ‘heavy’ labelled males

1. H1

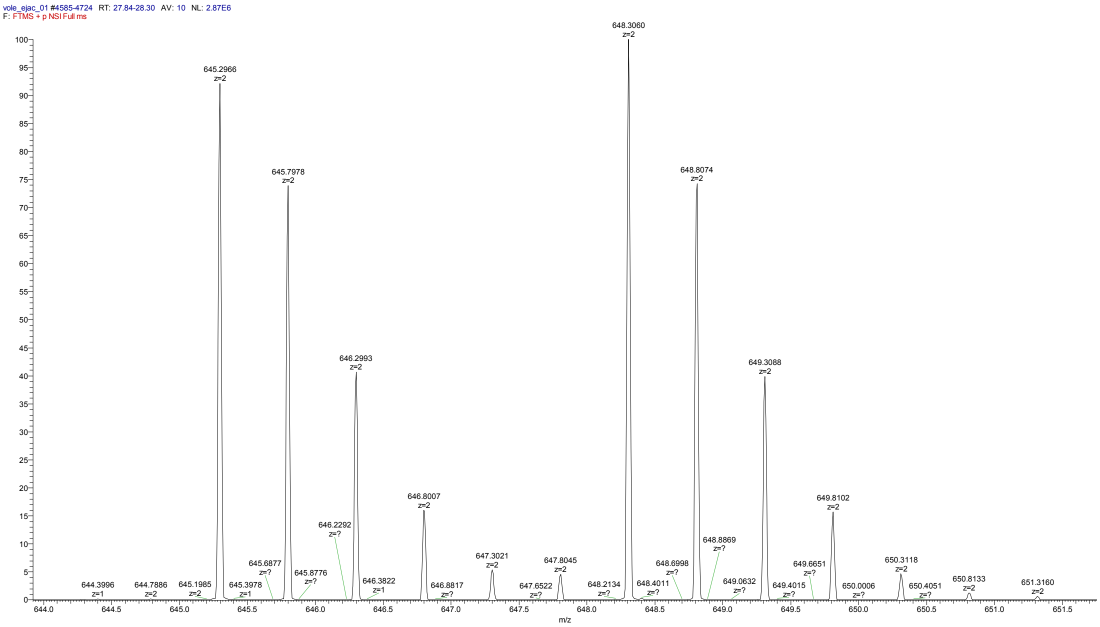

2. H2

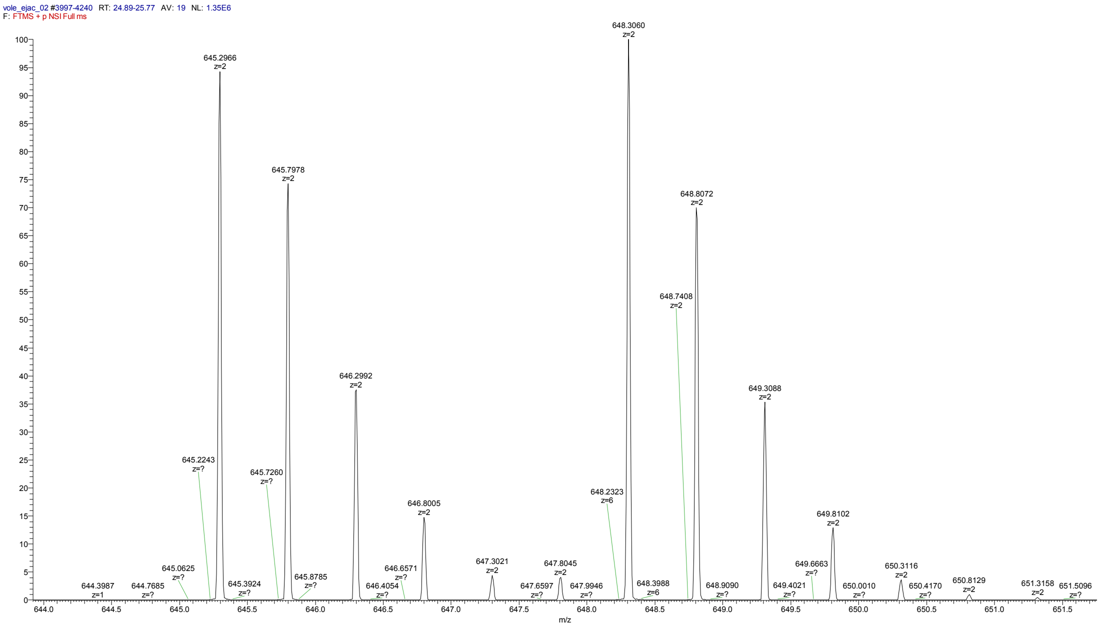

3. H3

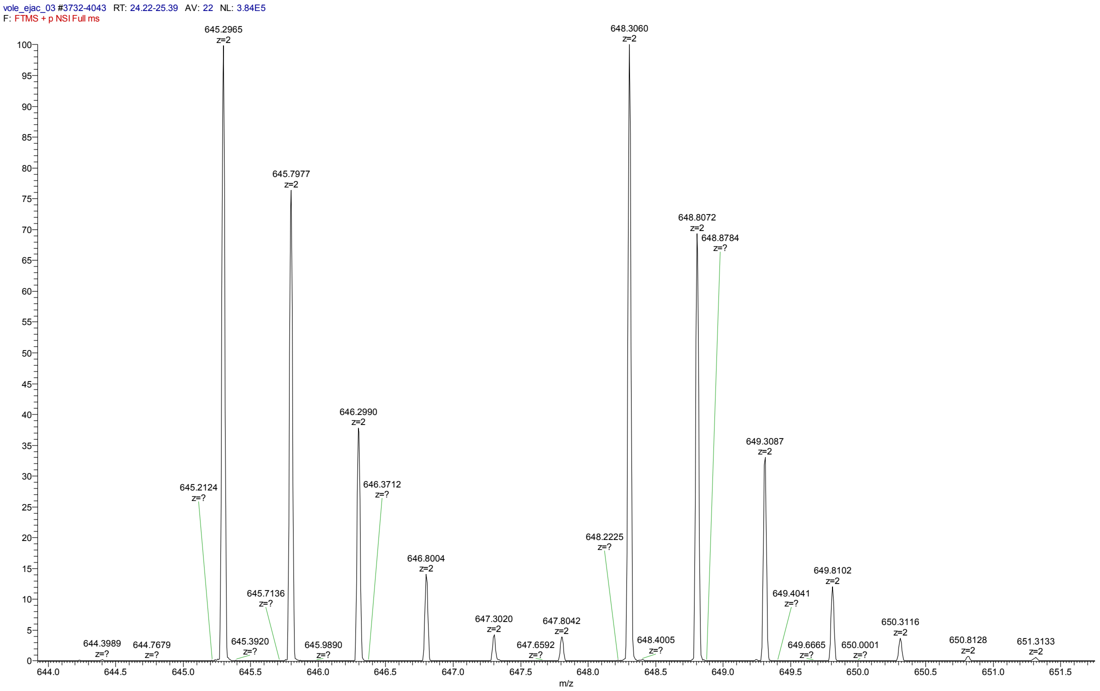
vole_ejac_03 #3732-4043 RT: 24.22-25.39 AV: 22 NL: 3.84E5
F: FTMS + p NSI Full ms
645.2965 z=2
648.3060 z=2
645.7977 z=2
648.8072 z=2
648.8784 z=?
646.2990 z=2
649.3087 z=2
645.2124 z=?
646.3712 z=?
648.2225 z=?
646.8004 z=2
649.8102 z=2
645.7136 z=?
649.4041 z=?
647.3020 z=2
647.8042 z=2
650.3116 z=2
644.3989 z=?
644.7679 z=?
645.3920 z=?
645.9890 z=?
647.6592 z=?
648.4005 z=?
649.6665 z=?
650.0001 z=?
650.8128 z=2
651.3133 z=2
m/z

4. H4

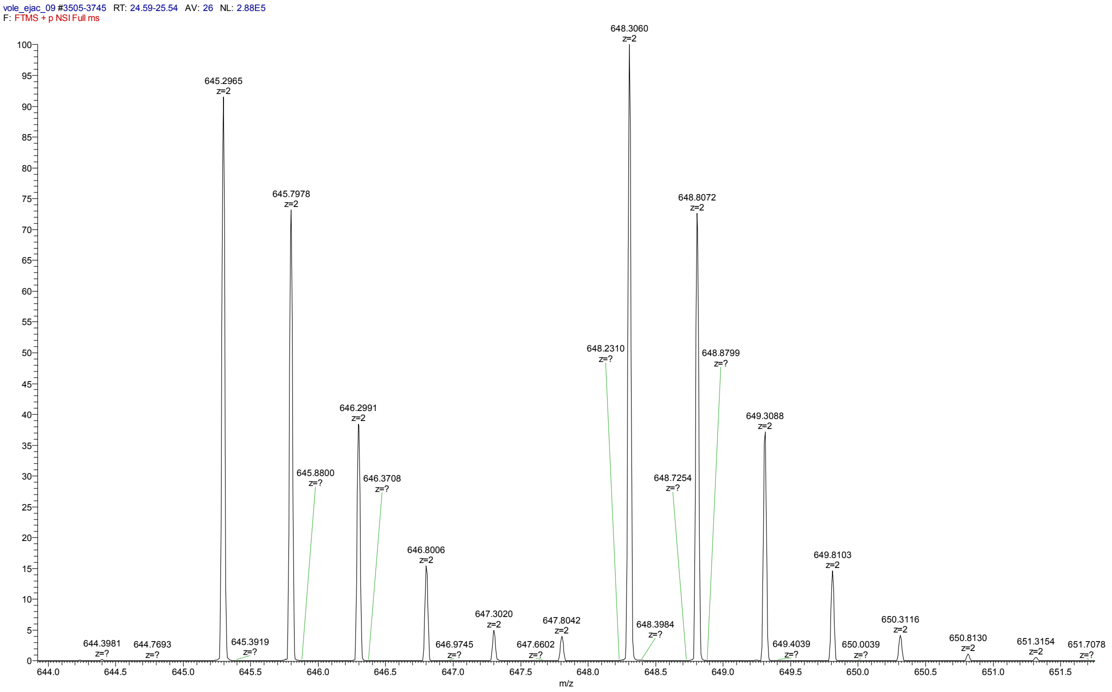
vole_ejac_09 #3505-3745 RT: 24.59-25.54 AV: 26 NL: 2.88E5
F: FTMS + p NSI Full ms
648.3060 z=2
645.2965 z=2
645.7978 z=2
648.8072 z=2
648.2310 z=?
648.8799 z=?
646.2991 z=2
649.3088 z=2
645.8800 z=?
646.3708 z=?
648.7254 z=?
646.8006 z=2
649.8103 z=2
647.3020 z=2
647.8042 z=2
650.3116 z=2
648.3984 z=?
644.3981 z=?
644.7693 z=?
645.3919 z=?
646.9745 z=?
647.6602 z=?
649.4039 z=?
650.0039 z=?
650.8130 z=2
651.3154 z=2
651.7078 z=?
m/z

B. SVS4 in ejaculates recovered following single copulations by ‘light’ males

1. L1

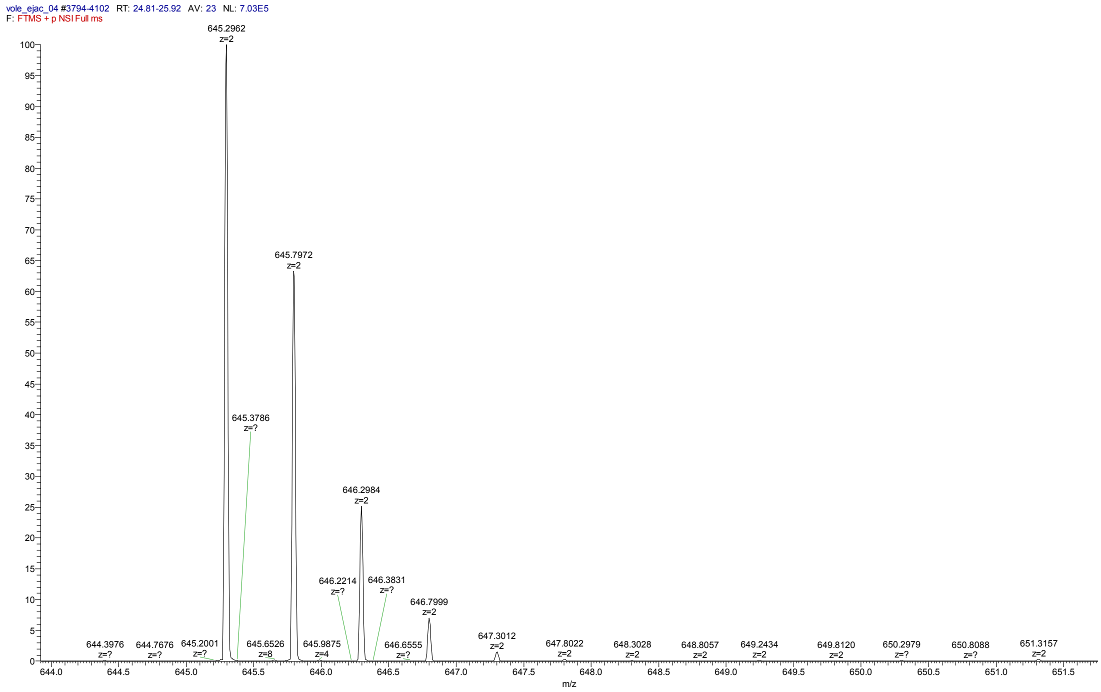

2. L2

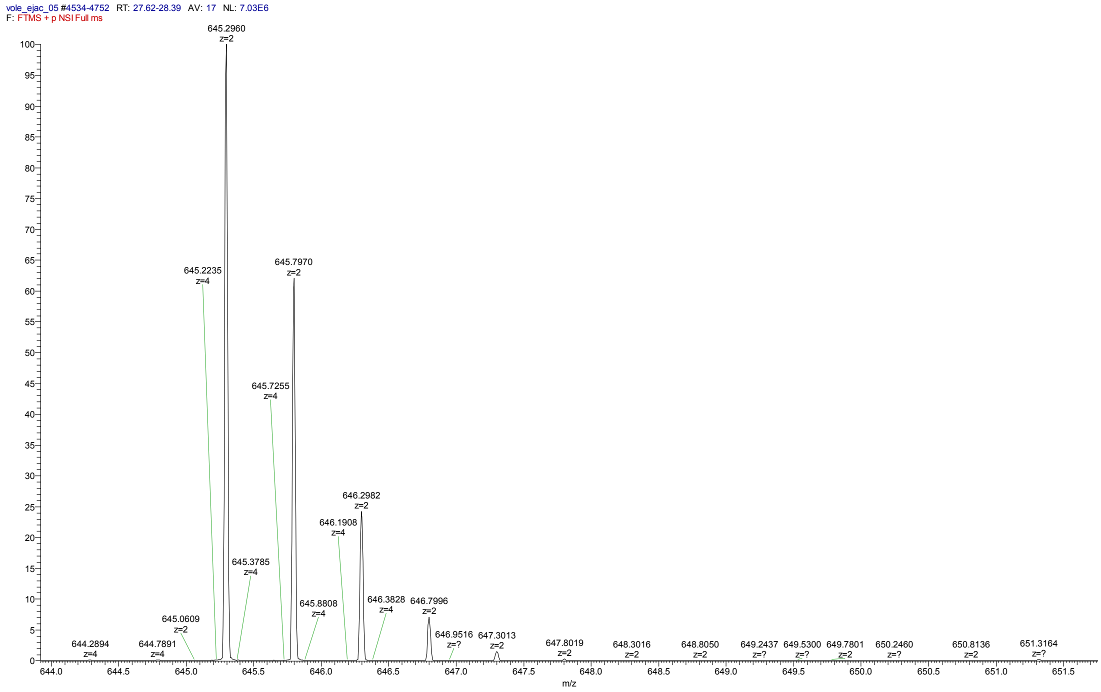

3. L3

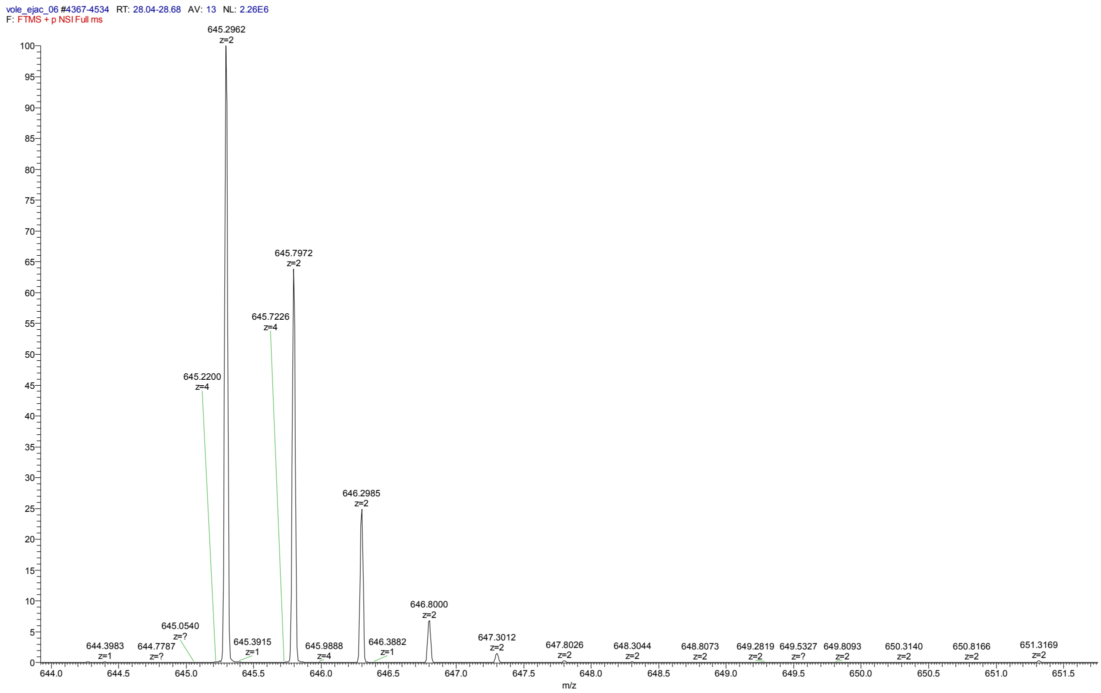
vole_ejac_06 #4367-4534 RT: 28.04-28.68 AV: 13 NL: 2.26E6
F: FTMS + p NSI Full ms
645.2962 z=2
645.7972 z=2
645.7226 z=4
645.2200 z=4
646.2985 z=2
646.8000 z=2
645.0540 z=?
647.3012 z=2
644.3983 z=1
644.7787 z=?
645.3915 z=1
645.9888 z=4
646.3882 z=1
647.8026 z=2
648.3044 z=2
648.8073 z=2
649.2819 z=2
649.5327 z=?
649.8093 z=2
650.3140 z=2
650.8166 z=2
651.3169 z=2
m/z

4. L4

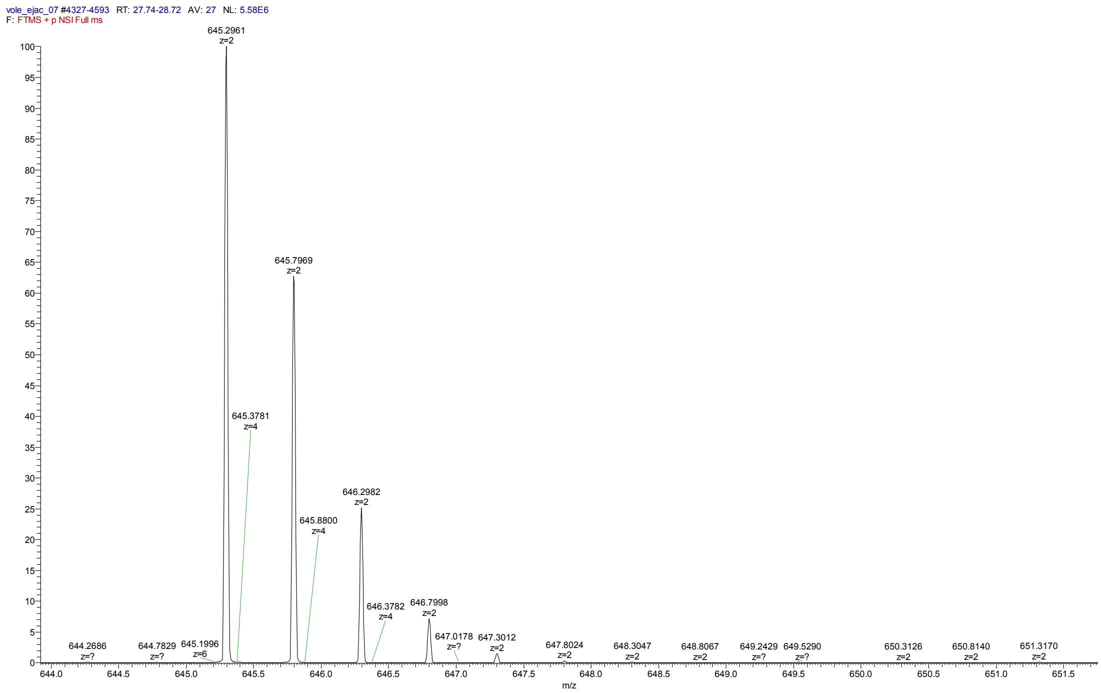
vole_ejac_07 #4327-4593 RT: 27.74-28.72 AV: 27 NL: 5.58E6
F: FTMS + p NSI Full ms
645.2961 z=2
645.7969 z=2
645.3781 z=4
646.2982 z=2
645.8800 z=4
646.3782 z=4
646.7998 z=2
647.0178 z=?
647.3012 z=2
644.2686 z=?
644.7829 z=?
645.1996 z=6
647.8024 z=2
648.3047 z=2
648.8067 z=2
649.2429 z=?
649.5290 z=?
650.3126 z=2
650.8140 z=2
651.3170 z=2
m/z

5. L5

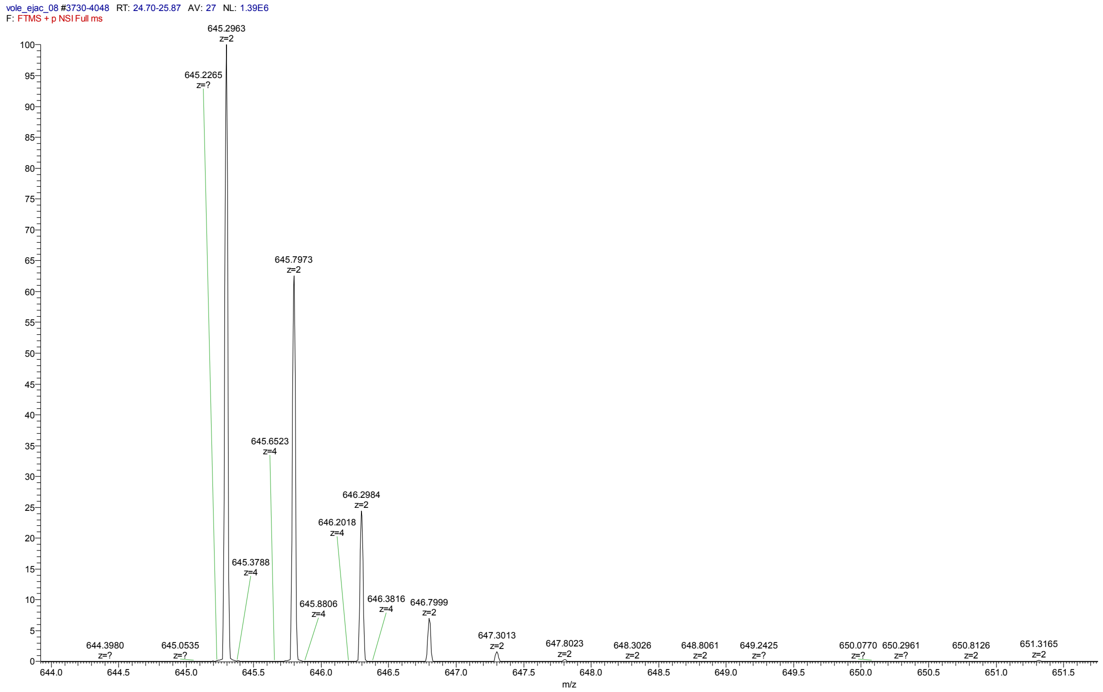
vole_ejac_08 #3730-4048 RT: 24.70-25.87 AV: 27 NL: 1.39E6
F: FTMS + p NSI Full ms
645.2963 z=2
645.2265 z=?
645.7973 z=2
645.6523 z=4
646.2984 z=2
646.2018 z=4
645.3788 z=4
645.8806 z=4
646.3816 z=4
646.7999 z=2
644.3980 z=?
645.0535 z=?
647.3013 z=2
647.8023 z=2
648.3026 z=2
648.8061 z=2
649.2425 z=?
650.0770 z=?
650.2961 z=?
650.8126 z=2
651.3165 z=2
m/z

6. L6

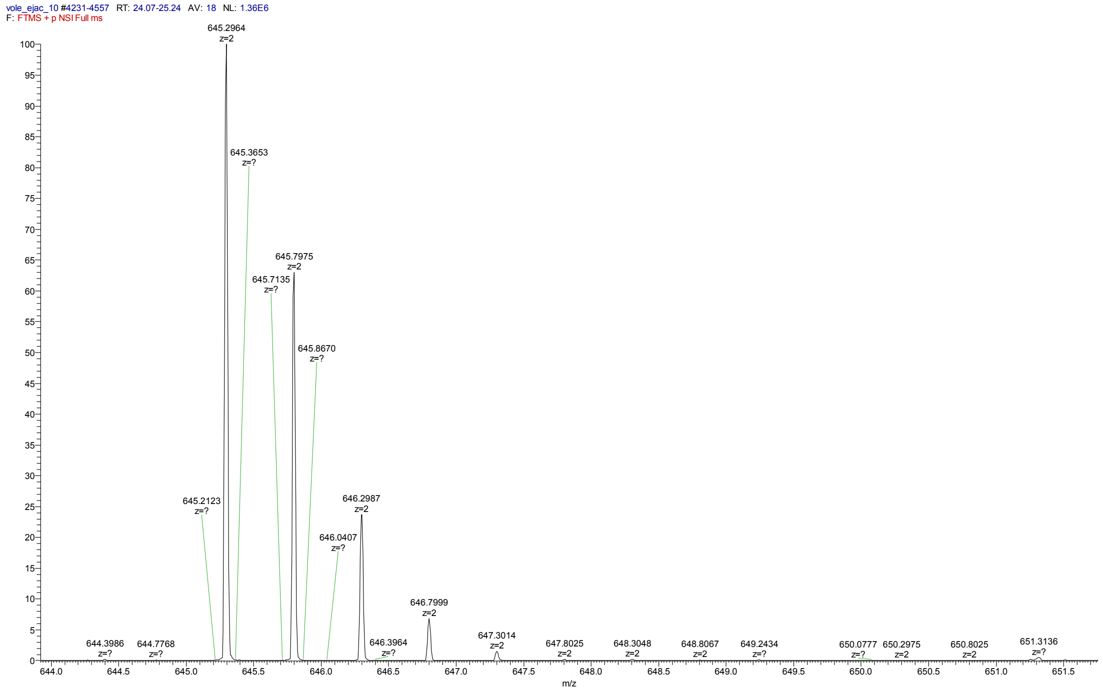
vole_ejac_10 #4231-4557 RT: 24.07-25.24 AV: 18 NL: 1.36E6
F: FTMS + p NSI Full ms
645.2964 z=2
645.3653 z=?
645.7975 z=2
645.7135 z=?
645.8670 z=?
645.2123 z=?
646.2987 z=2
646.0407 z=?
646.7999 z=2
644.3986 z=?
644.7768 z=?
646.3964 z=?
647.3014 z=2
647.8025 z=2
648.3048 z=2
648.8067 z=2
649.2434 z=?
650.0777 z=?
650.2975 z=2
650.8025 z=2
651.3136 z=?
m/z

7. L7

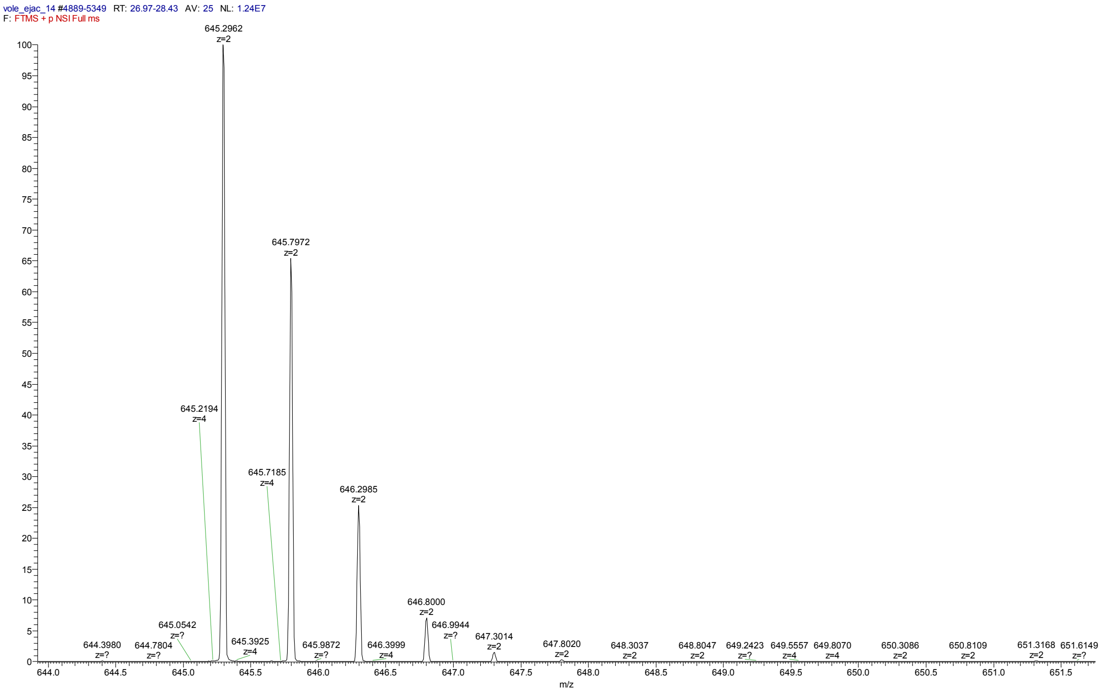

vole_ejac_14 #4889-5349 RT: 26.97-28.43 AV: 25 NL: 1.24E7
F: FTMS + p NSI Full ms
645.2962 z=2
645.7972 z=2
645.2194 z=4
645.7185 z=4
646.2985 z=2
646.8000 z=2
645.0542 z=?
646.9944 z=?
645.3925 z=4
647.3014 z=2
644.3980 z=?
644.7804 z=?
645.9872 z=?
646.3999 z=4
647.8020 z=2
648.3037 z=2
648.8047 z=2
649.2423 z=?
649.5557 z=4
649.8070 z=4
650.3086 z=2
650.8109 z=2
651.3168 z=2
651.6149 z=?
m/z

8. L8

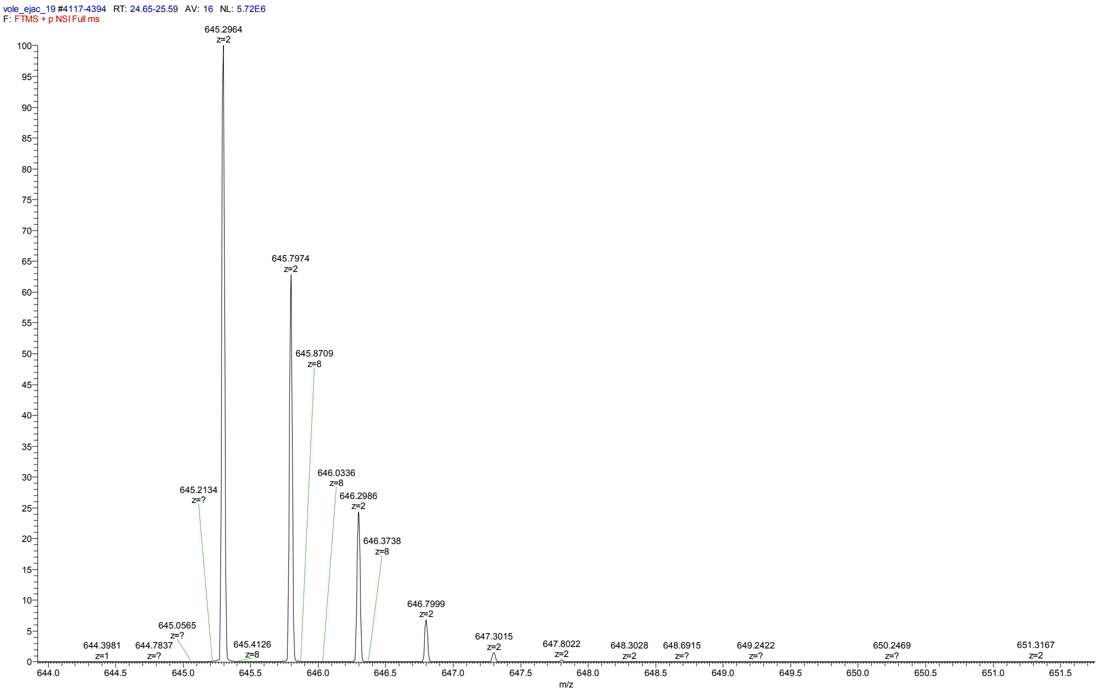

vole_ejac_19 #4117-4394 RT: 24.65-25.59 AV: 16 NL: 5.72E6
F: FTMS + p NSI Full ms
645.2964 z=2
645.7974 z=2
645.8709 z=8
646.0336 z=8
645.2134 z=?
646.2986 z=2
646.3738 z=8
646.7999 z=2
645.0565 z=?
644.3981 z=1
644.7837 z=?
645.4126 z=8
647.3015 z=2
647.8022 z=2
648.3028 z=2
648.6915 z=?
649.2422 z=?
650.2469 z=?
651.3167 z=2
m/z

9. L9

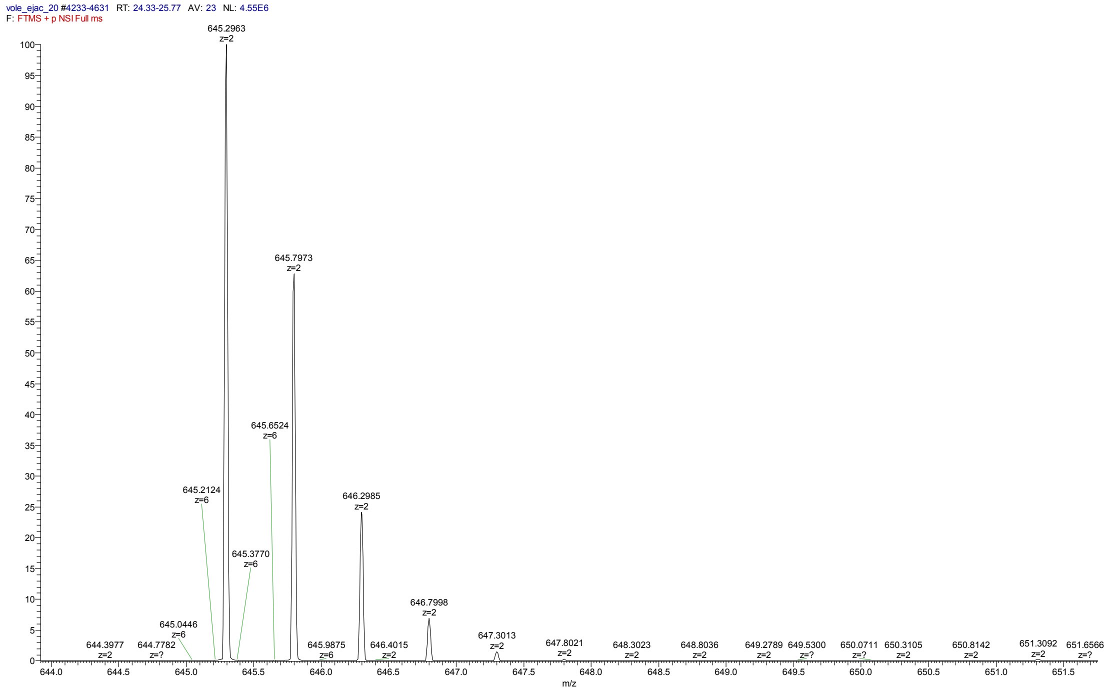
vole_ejac_20 #4233-4631 RT: 24.33-25.77 AV: 23 NL: 4.55E6
F: FTMS + p NSI Full ms
645.2963 z=2
645.7973 z=2
645.6524 z=6
645.2124 z=6
646.2985 z=2
645.3770 z=6
646.7998 z=2
645.0446 z=6
647.3013 z=2
644.3977 z=2
644.7782 z=?
645.9875 z=6
646.4015 z=2
647.8021 z=2
648.3023 z=2
648.8036 z=2
649.2789 z=2
649.5300 z=?
650.0711 z=?
650.3105 z=2
650.8142 z=2
651.3092 z=2
651.6566 z=?
m/z

10. L10

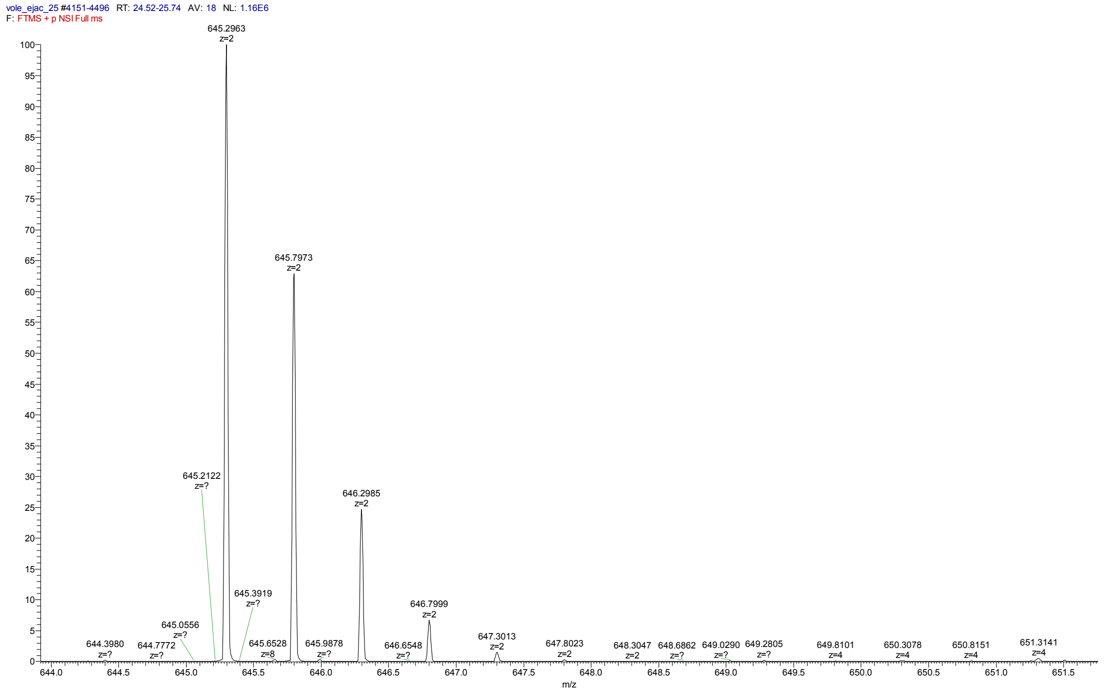
vole_ejac_25 #4151-4496 RT: 24.52-25.74 AV: 18 NL: 1.16E6
F: FTMS + p NSI Full ms
645.2963 z=2
645.7973 z=2
645.2122 z=?
646.2985 z=2
645.3919 z=?
646.7999 z=2
645.0556 z=?
647.3013 z=2
644.3980 z=?
644.7772 z=?
645.6528 z=8
645.9878 z=?
646.6548 z=?
647.8023 z=2
648.3047 z=2
648.6862 z=?
649.0290 z=?
649.2805 z=?
649.8101 z=4
650.3078 z=4
650.8151 z=4
651.3141 z=4
m/z

11. L11

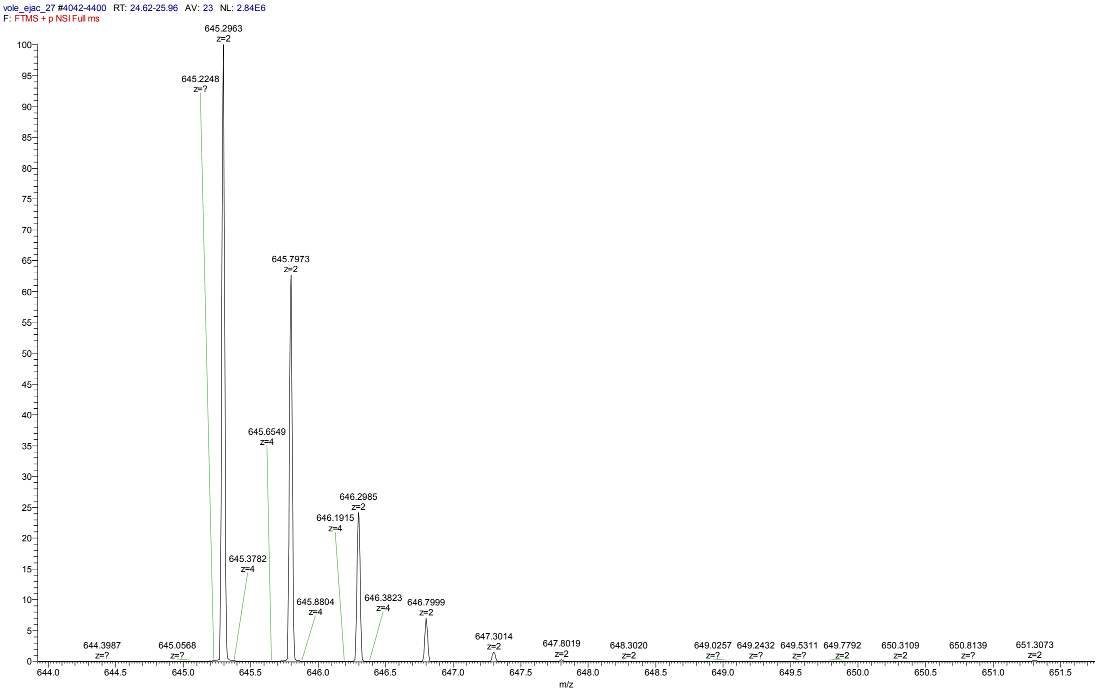
vole_ejac_27 #4042-4400 RT: 24.62-25.96 AV: 23 NL: 2.84E6
F: FTMS + p NSI Full ms
645.2963 z=2
645.2248 z=?
645.7973 z=2
645.6549 z=4
646.2985 z=2
646.1915 z=4
645.3782 z=4
645.8804 z=4
646.3823 z=4
646.7999 z=2
647.3014 z=2
644.3987 z=?
645.0568 z=?
647.8019 z=2
648.3020 z=2
649.0257 z=?
649.2432 z=?
649.5311 z=?
649.7792 z=2
650.3109 z=2
650.8139 z=?
651.3073 z=2
m/z

12. L12

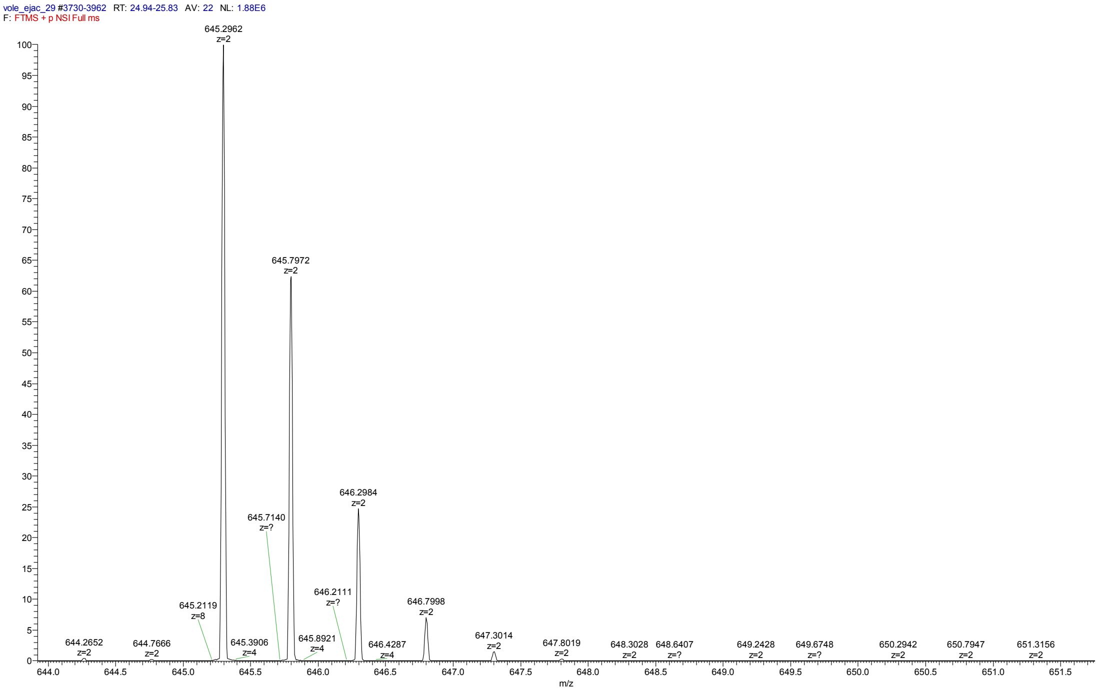
vole_ejac_29 #3730-3962 RT: 24.94-25.83 AV: 22 NL: 1.88E6
F: FTMS + p NSI Full ms
645.2962 z=2
645.7972 z=2
646.2984 z=2
645.7140 z=?
646.2111 z=?
645.2119 z=8
646.7998 z=2
644.2652 z=2
644.7666 z=2
645.3906 z=4
645.8921 z=4
646.4287 z=4
647.3014 z=2
647.8019 z=2
648.3028 z=2
648.6407 z=?
649.2428 z=2
649.6748 z=?
650.2942 z=2
650.7947 z=2
651.3156 z=2
m/z

13. L13

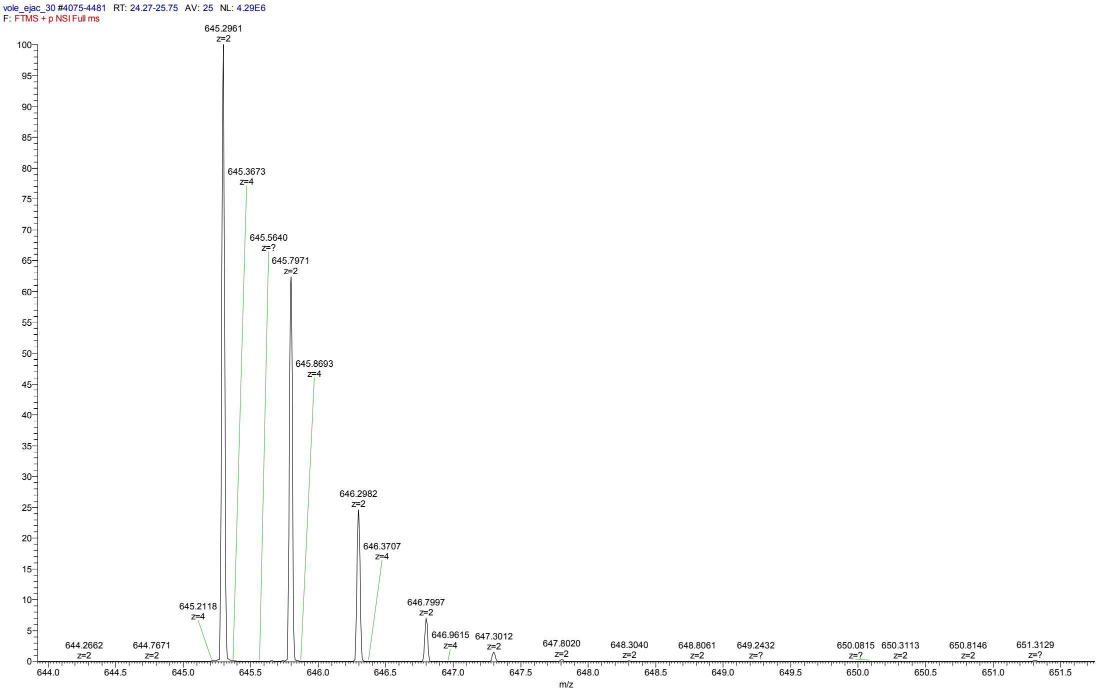
vole_ejac_30 #4075-4481 RT: 24.27-25.75 AV: 25 NL: 4.29E6
F: FTMS + p NSI Full ms
645.2961 z=2
645.3673 z=4
645.5640 z=?
645.7971 z=2
645.8693 z=4
646.2982 z=2
646.3707 z=4
645.2118 z=4
646.7997 z=2
646.9615 z=4
647.3012 z=2
644.2662 z=2
644.7671 z=2
647.8020 z=2
648.3040 z=2
648.8061 z=2
649.2432 z=?
650.0815 z=?
650.3113 z=2
650.8146 z=2
651.3129 z=?
m/z

14. L14

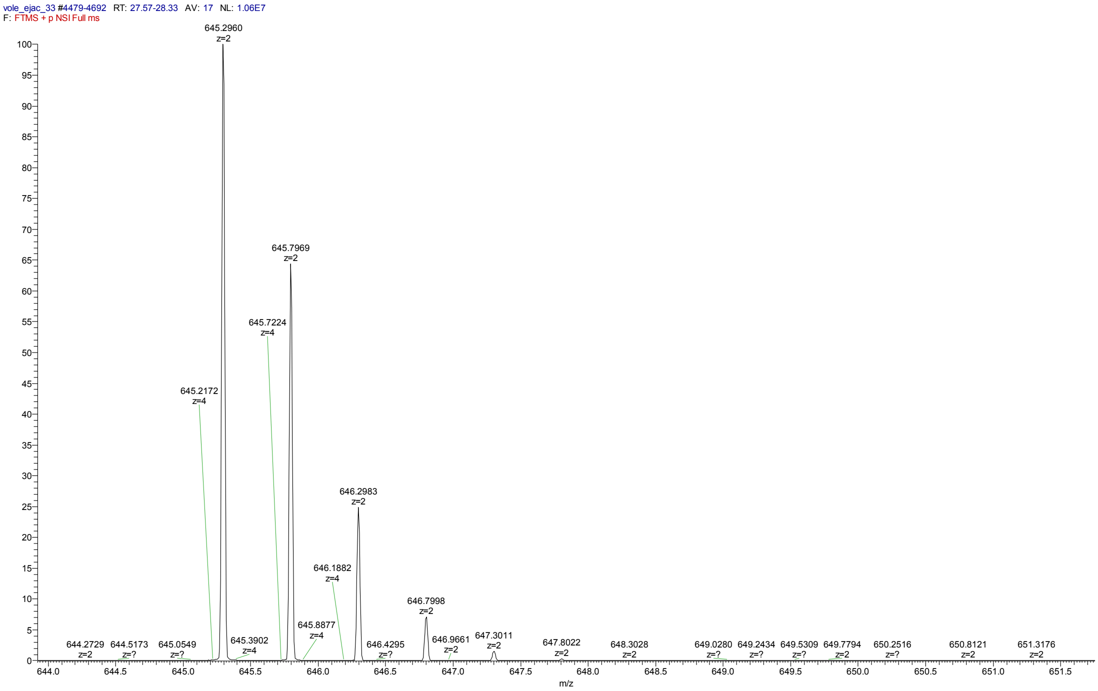
vole_ejac_33 #4479-4692 RT: 27.57-28.33 AV: 17 NL: 1.06E7
F: FTMS + p NSI Full ms
645.2960 z=2
645.7969 z=2
645.7224 z=4
645.2172 z=4
646.2983 z=2
646.1882 z=4
646.7998 z=2
645.8877 z=4
644.2729 z=2
644.5173 z=?
645.0549 z=?
645.3902 z=4
646.4295 z=?
646.9661 z=2
647.3011 z=2
647.8022 z=2
648.3028 z=2
649.0280 z=?
649.2434 z=?
649.5309 z=?
649.7794 z=2
650.2516 z=?
650.8121 z=2
651.3176 z=2
m/z

Table S1. Influence of dislodgement or retention of the first male's mating plug on the absolute number of sperm from competing males that reach the uterus. Results are shown for linear mixed models fit by maximum likelihood, testing the effect of plug dislodgement or retention on the absolute number of sperm recovered from the second male (A) and from the first male (B & C). Mean values are shown in Fig. S3A. The analysis in (A) and (B) includes 17 double copulations, with 17 males in role 2 (mating second) and 11 in role 1 (mating first). Male 1 ID is included as a random factor and sperm number when the focal male mated alone is included as a covariate. However, covariate data were not available for all males mating in role 1. Hence the analysis of first male sperm numbers in (B) reduces to 13 double copulations by 8 males. (C) Excludes one case in which no sperm from a male mating first were found in the uterus, leaving 12 double copulations by 7 males.

**Absolute number of sperm recovered from double mating**	X_1^2	*P*
**A) Second male**		
Retention of first male's mating plug	4.09	**0.04***
Sperm number in uterus when focal male mated alone	6.76	**<0.01****
**B) First male**		
Retention of first male's mating plug	2.71	0.10
Sperm number in uterus when focal male mated alone	2.63	0.10
**C) First male (excluding one for which no sperm recovered)**		
Retention of first male's mating plug	0.73	0.39
Sperm number in uterus when focal male mated alone	6.16	**0.01***

Table S2. Copulatory behaviours in first versus second mating role. Comparisons of copulatory behaviour when the mating male is the female's first versus second mate. A) Copulation duration (from first intromission to ejaculation) was significantly longer for the second male to mate; B) the second male to mate performed significantly more intromissions per copulation, but C) the intromission rate (number of intromissions divided by copulation duration) and D) copulation latency (from introduction to initiation of copulation) were not significantly different for the first and second copulation. Results are shown for linear mixed models fit by REML, using data for 60 copulations (40 with males mating first or alone, and 20 with males mating second in a double copulation), by 24 males with 40 females. The analysis includes male and female ID as random factors. Copulation duration is log transformed. Intromission rate and copulation latency are square root transformed.

**Factor**			Mean (± SE)	
	X_1^2	*P*	First male	Second male
**A) Copulation duration**			1181 (± 115) s	1837 (± 225) s
Mating role	12.67	**<0.001*** **		
**B) Number of intromissions**			33 (± 2.5)	59 (± 6.7)
Mating role	26.14	**<0.001*** **		
**C) Intromission rate**			2.17 (± 0.2) /m	2.11 (± 0.2)/m
Mating role	0.18	NS		
**D) Copulation latency**			249 (± 44) s	256 (± 40) s
Mating role	0.50	NS		

Table S3. Copulatory behaviour doesn't predict plug retention. Following sequential copulations by two males with the same female, the retention of mating plug material from the first male within the female reproductive tract was not predicted by copulatory behaviour in the first or second copulations: A) copulation duration (first intromission to ejaculation), B) number of intromissions per copulation, and C) intromission rate (number of intromissions divided by copulation duration). Results are shown for linear mixed models fit by REML, using data for 17 double copulations, with 17 males in the second mating role and 11 in the first mating role. The analysis includes the first male's ID as a random factor. Mating duration (all males) and intromission number (second males) are log transformed.

**Factor**	**First male**		**Second male**	
	X_1^2	*P*	X_1^2	*P*
**A) Copulation duration**				
Plug retention	0.11	NS	0.42	NS
**B) Number of intromissions**				
Plug retention	0.90	NS	0.48	NS
**C) Intromission rate**				
Plug retention	0.19	NS	1.68	NS

Table S4. Seminal vesicles mass predicts mating plug mass. When subject males mated in either first (in single copulations) or second (in double copulations) mating roles, the mass of the mating plug recovered from the uterus immediately after ejaculation was strongly predicted by the mass of the mating male's seminal vesicles, but not by their mating role (first or second to mate). Body mass is included as a covariate. Results are shown for linear mixed models fit by REML, for 18 males mating both first (in single copulations) and second (in double copulations) mating roles. Plug mass data excludes 4 cases where proteomic analysis revealed that the recovered plug originated from more than one. Male ID is included as a random factor. Plug mass is log transformed.

**Factor**	X_1^2	*P*
**Mating plug mass**		
Seminal vesicle mass	12.2	**<0.001*** **
Mating role (first or second to mate)	0.02	NS
Body mass	0.42	NS

Table S5. Selection of proteins used to quantify relative sperm numbers from competing males. To calculate the number of sperm from 'heavy' and 'light' labelled males in the uterus following double copulations, a set of sperm derived proteins was identified. Initially, data were recovered from 46 proteins from recovered ejaculates. Those that were unlikely to be male-specific were first eliminated by analysing four ejaculates from heavy labelled males that had each mated once with a different (unlabelled) female. Proteins were only retained for analysis if their abundance from the heavy labelled male was greater than 90% (median proportion over four ejaculates >0.9). Sperm proteins were then identified based on two criteria. First, proteins that were significantly correlated with the number of sperm counted in each sample. To account for multiple testing a Bonferroni correction was applied, with an adjusted significance level of 0.001. Second, remaining proteins were individually examined to check that they were likely to be found in sperm. The remaining 21 proteins were used to calculate sperm numbers from competing males (see Table S7 for a list of peptides used).

Protein	Male origin	Correlates with sperm number	Sperm-related	Used for calculation
14-3-3 protein zeta/delta	✗	--	--	No
2-oxoglutarate dehydrogenase, mitochondrial	✓	✓	✓	Yes
61 kDa heat shock protein, mitochondrial	✗	--	--	No
Aconitate hydratase, mitochondrial	✓	✓	✓	Yes
ADP/ATP translocase 4	✓	✓	✓	Yes
A-kinase anchor protein 3	✓	✓	✓	Yes
A-kinase anchor protein 4	✓	✓	✓	Yes

Alpha-enolase	✗	--	--	No
Annexin A6	✓	✗	--	No
ATP synthase subunit alpha, mitochondrial	✓	✓	✓	Yes
ATP synthase subunit beta, mitochondrial	✓	✓	✓	Yes
Calicin	✓	✓	✓	Yes
Calmodulin	✗	--	--	No
Citrate synthase, mitochondrial	✓	✓	✓	Yes
Cytochrome b-c1 complex subunit 2, mitochondrial	✓	✓	✓	Yes
Cytosol aminopeptidase	✓	✓	✗	No
Cytosolic 5'-nucleotidase 1B	✗	--	--	No
Dihydrolipoyllysine-residue acetyltransferase component of pyruvate dehydrogenase complex, mitochondrial	✓	✓	✓	Yes
Dynein heavy chain 8, axonemal	✓	✓	✓	Yes
F-actin-capping protein subunit alpha-3	✓	✓	✓	Yes
Fructose-bisphosphate aldolase A	✗	--	--	No
Glucose-6-phosphate isomerase	✗	--	--	No
Glutathione S-transferase Mu 6	✓	✓	✗	No
Heat shock cognate 71 kDa protein	✗	--	--	No
L-lactate dehydrogenase A chain	✗	--	--	No
Malate dehydrogenase, mitochondrial	✗	--	--	No
Outer dense fiber protein 2	✓	✓	✓	Yes
Outer dense fiber protein 3	✗	--	--	No
Phosphoglycerate kinase 1	✗	--	--	No
Phospholipid hydroperoxide glutathione peroxidase, mitochondrial	✓	✓	✓	Yes
Protein-glutamine gamma-glutamyltransferase 4	✓	✓	✗	No
Pyruvate dehydrogenase E1 component subunit beta, mitochondrial	✓	✓	✓	Yes
Pyruvate kinase isozymes M1/M3	✗	--	--	No
Ropporin-1	✓	✓	✓	Yes

Serotransferrin	✗	--	--	No
Serum albumin	✗	--	--	No
Sodium/potassium-transporting ATPase subunit alpha-4	✓	✓	✓	Yes
Succinate dehydrogenase [ubiquinone] flavoprotein subunit, mitochondrial	✗	--	--	No
SVS4_CGrf SVS 4 Vole DNA	✓	✗	--	No
Testis-specific gene 10 protein	✗	--	--	No
Transitional endoplasmic reticulum ATPase	✗	--	--	No
Triosephosphate isomerase	✗	--	--	No
Tubulin alpha-3 chain	✓	✓	✓	Yes
Tubulin beta-2C chain	✓	✓	✓	Yes
Voltage-dependent anion-selective channel protein 2	✗	--	--	No
Voltage-dependent anion-selective channel protein 3	✓	✓	✓	Yes

Table S6. Lysine-containing peptides used to define proportion of sperm originating from heavy and light labelled males. From 21 proteins identified as sperm derived, 83 lysine containing peptides were used to calculate sperm numbers from competing males following double copulations. These were identified from a total of 93 available peptides by removing any identified as giving outlying values. Outliers were defined as more than 2 IQRs above the upper quartile or below the lower quartile. Peptides were excluded if they were identified as outliers in 5 or more of 20 samples analysed.

Protein	Peptide
2-oxoglutarate dehydrogenase, mitochondrial	DPAAAPATGNK
2-oxoglutarate dehydrogenase, mitochondrial	IEQLSPFPFDLLLK
Aconitate hydratase, mitochondrial	DLEDLQILIK
Aconitate hydratase, mitochondrial	IHETNLK
Aconitate hydratase, mitochondrial	LQLLEPFDK
Aconitate hydratase, mitochondrial	LTGSLSGWTSPK
Aconitate hydratase, mitochondrial	LTIQGLK
Aconitate hydratase, mitochondrial	VAGILTVK
Aconitate hydratase, mitochondrial	YDLLEK
ADP/ATP translocase 4	ASYFGAYDTVK
ADP/ATP translocase 4	DLLAGGVAAAVSK
ADP/ATP translocase 4	LGVDIGK
A-kinase anchor protein 3	DTTIATILLK
A-kinase anchor protein 3	EVVSDLIDSFMK
A-kinase anchor protein 4	DLIVSALMLIQYHLTQQAK
A-kinase anchor protein 4	EFADSISK
A-kinase anchor protein 4	GLMVYANQVASDMMVSVMK
A-kinase anchor protein 4	GYSVGDLLQEVMK
A-kinase anchor protein 4	KQLLDWLLANL

A-kinase anchor protein 4	LEMAASK
A-kinase anchor protein 4	LPEVSAK
A-kinase anchor protein 4	NLFNHGK
A-kinase anchor protein 4	NLHNITGVLMTDSDFVSAVK
A-kinase anchor protein 4	QFIDQLVESVMK
A-kinase anchor protein 4	QNAADIMEAMLK
A-kinase anchor protein 4	RLVSALLGEK
ATP synthase subunit alpha, mitochondrial	GMSLNLEPDNVGVVVFGNDK
ATP synthase subunit alpha, mitochondrial	LTELLK
ATP synthase subunit alpha, mitochondrial	RFNDGTDEK
ATP synthase subunit alpha, mitochondrial	TSIAIDTIINQK
ATP synthase subunit beta, mitochondrial	ILQDYK
ATP synthase subunit beta, mitochondrial	TVLIMELINNVAK
Calicin	YLFLAELFELK
Citrate synthase, mitochondrial	ALGFPLERPK
Citrate synthase, mitochondrial	EFALK
Citrate synthase, mitochondrial	SMSTDGLMK
Cytochrome b-c1 complex subunit 2, mitochondrial	AVAQGNLSSADVQAAK
Dihydrolipoyllysine-residue acetyltransferase component of pyruvate dehydrogenase complex, mitochondrial	DIDSFVPSK
Dihydrolipoyllysine-residue acetyltransferase component of pyruvate dehydrogenase complex, mitochondrial	GLETIASDVVSLASK
Dihydrolipoyllysine-residue acetyltransferase component of pyruvate dehydrogenase complex, mitochondrial	ISVNDFIIK
Dihydrolipoyllysine-residue acetyltransferase component of pyruvate dehydrogenase complex, mitochondrial	VFVSPLAK
Dihydrolipoyllysine-residue acetyltransferase component of pyruvate dehydrogenase complex, mitochondrial	YLEKPITMLL

F-actin-capping protein subunit alpha-3	FFDYQSK
F-actin-capping protein subunit alpha-3	SKWIFQVNPFLTQVTGR
Outer dense fiber protein 2	DFTMLQK
Outer dense fiber protein 2	ELLLQK
Outer dense fiber protein 2	KEELEEVAHELAETEHENTVLR
Outer dense fiber protein 2	LLLLLQDK
Outer dense fiber protein 2	LNQAHLEVQQLK
Outer dense fiber protein 2	NYEGMIDNYK
Outer dense fiber protein 2	QFQSQLADLQQLPDILK
Outer dense fiber protein 2	QTAEYSAFK
Outer dense fiber protein 2	SEEYAEQLHVQLADK
Outer dense fiber protein 2	VTDLVNQQQSLEEK
Outer dense fiber protein 2	YNQVVK
Phospholipid hydroperoxide glutathione peroxidase, mitochondrial	EFAAGYNVK
Phospholipid hydroperoxide glutathione peroxidase, mitochondrial	FLIDK
Phospholipid hydroperoxide glutathione peroxidase, mitochondrial	GMLGNAIK
Phospholipid hydroperoxide glutathione peroxidase, mitochondrial	QEPGSNQEIK
Phospholipid hydroperoxide glutathione peroxidase, mitochondrial	WNFTK
Pyruvate dehydrogenase E1 component subunit beta, mitochondrial	DFLIPIGK
Pyruvate dehydrogenase E1 component subunit beta, mitochondrial	DIIFAVK
Pyruvate dehydrogenase E1 component subunit beta, mitochondrial	TIRPMDIEAIEASVMK
Pyruvate dehydrogenase E1 component subunit beta, mitochondrial	VFLLGEEVAQYDGAYK
Pyruvate dehydrogenase E1 component subunit beta, mitochondrial	VVSPWNSEDAK
Ropporin-1	LIIHADELAQMWK
Ropporin-1	MLNYIEQEVIGPDGLIK
Tubulin alpha-3 chain	DVNAAIATIK
Tubulin alpha-3 chain	EDLAALEK
Tubulin alpha-3 chain	FDLMYAK

Tubulin alpha-3 chain	IHFPLATYAPVISAEK
Tubulin alpha-3 chain	LSVDYGK
Tubulin alpha-3 chain	QLFHPEQLITGK
Tubulin alpha-3 chain	VGINYQPPTVVPGGDLAK
Tubulin beta-2C chain	ALTVPELTQQMFDAK
Tubulin beta-2C chain	EVDEQMLNVQNK
Tubulin beta-2C chain	KLAVNMVPFPR
Tubulin beta-2C chain	NSSYFVEWIPNNVK
Voltage-dependent anion-selective channel protein 3	GYGFGMVK
Voltage-dependent anion-selective channel protein 3	LAEGLK
Voltage-dependent anion-selective channel protein 3	LSQNNFALGYK
Voltage-dependent anion-selective channel protein 3	VNNASLIGLGYTQTLRPGVK
Voltage-dependent anion-selective channel protein 3	WNTDNTLGTEISWENK

Table S7. Markers used for genotyping analysis.

Locus	GenBank accession	Repeat	Primer sequence 5′–3′
Cg16A3	EU285428	$(CT)_{22}$	F: TAACTGCCAAGGGTGATAGA R: TCCAAATCTTTTGACCTACCT
Cg2D3	EU285383	$(CT)_{22}(CA)_{13}$	F: TCCTTCCTCTTTATTCACTCC R: GAGCAACACTAAACGGATTC
Cg5E8	EU285398	$(GT)_{19}(GA)_{21}$	F: ATTTCATAAACGCCTCCTTC R: AGTTCAGTCAGTGGATCCTG
Cg17D5	EU285438	$(GA)_{11}N_2(GA)_7$	F: TTTTAGCCTCATAAATGGACA R: GCAGTAGATTTCGCTCACAC
Cg2A4	EU285381	$(GT)_{15}(GA)_{13}$	F: GGACTTGATAGGGGAGAAA R: CTCTTTCATCCTTGCTTGT
Cg13B8	EU285417	$(CT)_{20}(GT)_9$	F: GCCTAATGTTTTCTCTGTGC R: CACATGGAATGAGGTTCTTAC
